# Associations of Neighborhood Opportunity and Social Vulnerability With Trajectories of Childhood Body Mass Index and Obesity Among US Children

**DOI:** 10.1001/jamanetworkopen.2022.47957

**Published:** 2022-12-22

**Authors:** Izzuddin M. Aris, Wei Perng, Dana Dabelea, Amy M. Padula, Akram Alshawabkeh, Carmen M. Vélez-Vega, Judy L. Aschner, Carlos A. Camargo, Tamara J. Sussman, Anne L. Dunlop, Amy J. Elliott, Assiamira Ferrara, Yeyi Zhu, Christine L. M. Joseph, Anne Marie Singh, Tina Hartert, Ferdinand Cacho, Margaret R. Karagas, Tiffany North-Reid, Barry M. Lester, Nichole R. Kelly, Jody M. Ganiban, Su H. Chu, Thomas G. O’Connor, Rebecca C. Fry, Gwendolyn Norman, Leonardo Trasande, Bibiana Restrepo, Peter James, Emily Oken

**Affiliations:** 1 Division of Chronic Disease Research Across the Lifecourse, Department of Population Medicine, Harvard Medical School and Harvard Pilgrim Health Care Institute, Boston, Massachusetts; 2 Department of Epidemiology, Colorado School of Public Health, University of Colorado Anschutz Medical Campus, Aurora; 3 Lifecourse Epidemiology of Adiposity and Diabetes (LEAD) Center, University of Colorado Anschutz Medical Campus, Aurora; 4 Department of Pediatrics, University of Colorado Anschutz Medical Campus, Aurora; 5 Department of Obstetrics, Gynecology and Reproductive Sciences, University of California, San Francisco, San Francisco; 6 Department of Civil and Environmental Engineering, Northeastern University, Boston, Massachusetts; 7 UPR Medical Sciences Campus, University of Puerto Rico Graduate School of Public Health, San Juan; 8 Department of Pediatrics, Hackensack Meridian School of Medicine, Nutley, New Jersey; 9 Department of Pediatrics, Albert Einstein College of Medicine, New York, New York; 10 Department of Epidemiology, Harvard T.H. Chan School of Public Health, Boston, Massachusetts; 11 Channing Division of Network Medicine, Brigham and Women’s Hospital, Harvard Medical School, Boston, Massachusetts; 12 Department of Emergency Medicine, Massachusetts General Hospital, Boston; 13 Department of Psychiatry, Columbia University and New York State Psychiatric Institute, New York, New York; 14 Department of Gynecology and Obstetrics, Emory University School of Medicine, Atlanta, Georgia; 15 Avera Research Institute, Sioux Falls, South Dakota; 16 Department of Pediatrics, University of South Dakota School of Medicine, Sioux Falls; 17 Division of Research, Kaiser Permanente Northern California, Oakland; 18 Department of Public Health Sciences, Henry Ford Health System, Detroit, Michigan; 19 Division of Allergy, Immunology and Rheumatology, University of Wisconsin–Madison, Madison; 20 Department of Medicine, Vanderbilt University Medical Center, Nashville, Tennessee; 21 Department of Pediatrics, Vanderbilt University Medical Center, Nashville, Tennessee; 22 Department of Epidemiology, Dartmouth Geisel School of Medicine, Hanover, New Hampshire; 23 Department of Pediatrics, Warren Alpert Medical School of Brown University, Providence, Rhode Island; 24 Department of Counseling Psychology and Human Services, Prevention Science Institute, University of Oregon, Eugene; 25 Department of Psychological and Brain Sciences, George Washington University, Washington, District of Columbia; 26 Department of Psychiatry, University of Rochester, Rochester, New York; 27 Department of Environmental Sciences and Engineering, Gillings School of Global Public Health, University of North Carolina, Chapel Hill; 28 Institute for Environmental Health Sciences, Wayne State University School of Medicine, Detroit, Michigan; 29 Department of Obstetrics and Gynecology, Wayne State University School of Medicine, Detroit, Michigan; 30 Department of Pediatrics, New York University Grossman School of Medicine, New York; 31 Department of Pediatrics, University of California Davis School of Medicine, Sacramento; 32 MIND Institute, University of California Davis, Sacramento, California; 33 Department of Environmental Health, Harvard T.H. Chan School of Public Health, Boston, Massachusetts; 34 Department of Nutrition, Harvard T.H. Chan School of Public Health, Boston, Massachusetts

## Abstract

**Importance:**

Physical and social neighborhood attributes may have implications for children’s growth and development patterns. The extent to which these attributes are associated with body mass index (BMI) trajectories and obesity risk from childhood to adolescence remains understudied.

**Objective:**

To examine associations of neighborhood-level measures of opportunity and social vulnerability with trajectories of BMI and obesity risk from birth to adolescence.

**Design, Setting, and Participants:**

This cohort study used data from 54 cohorts (20 677 children) participating in the Environmental Influences on Child Health Outcomes (ECHO) program from January 1, 1995, to January 1, 2022. Participant inclusion required at least 1 geocoded residential address and anthropometric measure (taken at the same time or after the address date) from birth through adolescence. Data were analyzed from February 1 to June 30, 2022.

**Exposures:**

Census tract–level Child Opportunity Index (COI) and Social Vulnerability Index (SVI) linked to geocoded residential addresses at birth and in infancy (age range, 0.5-1.5 years), early childhood (age range, 2.0-4.8 years), and mid-childhood (age range, 5.0-9.8 years).

**Main Outcomes and Measures:**

BMI (calculated as weight in kilograms divided by length [if aged <2 years] or height in meters squared) and obesity (age- and sex-specific BMI ≥95th percentile). Based on nationwide distributions of the COI and SVI, Census tract rankings were grouped into 5 categories: very low (<20th percentile), low (20th percentile to <40th percentile), moderate (40th percentile to <60th percentile), high (60th percentile to <80th percentile), or very high (≥80th percentile) opportunity (COI) or vulnerability (SVI).

**Results:**

Among 20 677 children, 10 747 (52.0%) were male; 12 463 of 20 105 (62.0%) were White, and 16 036 of 20 333 (78.9%) were non-Hispanic. (Some data for race and ethnicity were missing.) Overall, 29.9% of children in the ECHO program resided in areas with the most advantageous characteristics. For example, at birth, 26.7% of children lived in areas with very high COI, and 25.3% lived in areas with very low SVI; in mid-childhood, 30.6% lived in areas with very high COI and 28.4% lived in areas with very low SVI. Linear mixed-effects models revealed that at every life stage, children who resided in areas with higher COI (vs very low COI) had lower mean BMI trajectories and lower risk of obesity from childhood to adolescence, independent of family sociodemographic and prenatal characteristics. For example, among children with obesity at age 10 years, the risk ratio was 0.21 (95% CI, 0.12-0.34) for very high COI at birth, 0.31 (95% CI, 0.20-0.51) for high COI at birth, 0.46 (95% CI, 0.28-0.74) for moderate COI at birth, and 0.53 (95% CI, 0.32-0.86) for low COI at birth. Similar patterns of findings were observed for children who resided in areas with lower SVI (vs very high SVI). For example, among children with obesity at age 10 years, the risk ratio was 0.17 (95% CI, 0.10-0.30) for very low SVI at birth, 0.20 (95% CI, 0.11-0.35) for low SVI at birth, 0.42 (95% CI, 0.24-0.75) for moderate SVI at birth, and 0.43 (95% CI, 0.24-0.76) for high SVI at birth. For both indices, effect estimates for mean BMI difference and obesity risk were larger at an older age of outcome measurement. In addition, exposure to COI or SVI at birth was associated with the most substantial difference in subsequent mean BMI and risk of obesity compared with exposure at later life stages.

**Conclusions and Relevance:**

In this cohort study, residing in higher-opportunity and lower-vulnerability neighborhoods in early life, especially at birth, was associated with a lower mean BMI trajectory and a lower risk of obesity from childhood to adolescence. Future research should clarify whether initiatives or policies that alter specific components of neighborhood environment would be beneficial in preventing excess weight in children.

## Key Points

**Question:**

Is there an association between neighborhood-level measures of opportunity and social vulnerability in early life and trajectories of body mass index (BMI) and obesity risk from birth to adolescence?

**Findings:**

In this cohort study including 20 677 US children, residence in neighborhoods with higher-opportunity or lower social vulnerability in early life, especially at birth, was associated with a lower mean BMI trajectory and a lower risk of obesity from childhood to adolescence.

**Meaning:**

These findings suggest that residence in higher-opportunity neighborhoods in early life may promote maintenance of favorable BMI patterns during childhood.

## Teaser

This cohort study uses data from the Environmental Influences on Child Health Outcomes program to examine the associations of neighborhood-level measures of opportunity and social vulnerability with trajectories of body mass index and obesity risk among US children from birth to adolescence.

## Introduction

The quality of neighborhoods in which children reside has been increasingly recognized as an important factor associated with health across the lifecourse.^1,2^ Compared with adults, children may be particularly vulnerable to adverse neighborhood conditions, with consequences for lifelong health. For example, residence in a neighborhood with high rates of poverty and/or crime has been associated with increased risk of high body mass index (BMI) or obesity in childhood,^3,4^ which is a risk factor for chronic disease, such as type 2 diabetes, in adulthood.^5^ Such studies highlight the potential importance of neighborhood contexts as factors associated with high childhood BMI. However, most studies examining these questions have been cross-sectional in study design or focused on singular socioeconomic indicators of neighborhood disadvantage, such as rates of low household income or unemployment.^6,7,8^ Moreover, few studies have examined the extent to which neighborhood conditions during early life—at birth, in infancy, or in early childhood, which are key life stages that lay the foundation for long-term health outcomes^9^—are associated with BMI from childhood to adolescence. Even fewer studies have attempted to ascertain the sensitive periods of exposure to neighborhood conditions by comparing the consequences of exposures during multiple life stages.

An increasing body of research has begun to use Census-derived indices of neighborhood conditions as proxies for the specific physical or social features hypothesized to be etiologically relevant to a range of health outcomes. Two examples include the Child Opportunity Index (COI)^10^ and the Social Vulnerability Index (SVI).^11^ The COI is a multidimensional surveillance tool incorporating both traditional (eg, median household income) and novel (eg, access to healthy food choices or green space, walkability, and toxic exposures) attributes of neighborhood conditions that have been found to be associated with adolescents’ cardiometabolic health.^12^ The SVI is a relative measurement of neighborhood vulnerability and disadvantage based on 15 social factors (eg, socioeconomic status and household composition) that have been reported to be associated with adult obesity and cardiovascular disease.^13,14^ However, few studies have examined the extent to which these neighborhood indices may be associated with BMI trajectories and protection from obesity from childhood to adolescence and whether these indices are equally or differentially associated with BMI and obesity risk trajectories.

To address these knowledge gaps, we analyzed data from cohorts participating in the Environmental Influences on Child Health Outcomes (ECHO) program, which collected repeated measures of both residential addresses and children’s growth across distinct childhood life stages in a racially, ethnically, and geographically diverse population. We hypothesized that children residing in higher-opportunity or less vulnerable neighborhoods would have lower mean BMI and lower risk of obesity from childhood to adolescence. Because previous studies^15,16,17^ reported that later age periods were more sensitive than earlier periods with regard to an association with health outcomes, we also hypothesized that the effect estimates would be more substantial for COI or SVI exposure later in childhood than at birth or in infancy.

## Methods

### Study Population

ECHO is a large collaborative consortium comprising individual cohorts of children across the US; the consortium had already begun enrolling participants with the goal of investigating how environmental exposures in early life, including physical, chemical, social, behavioral, biological, natural, and built environments, were associated with children’s health and development. As detailed elsewhere,^18,19^ investigators of participating cohorts implemented the ECHO-wide cohort data collection protocol, which specifies the data elements for new or ongoing data collection as well as extant data to be uploaded onto an ECHO-wide cohort data platform. For the current cohort study, we used extant data previously collected from individual cohorts; these data were already harmonized and shared on the ECHO data platform. We included participants from any cohort in the analytic sample if they had at least 1 geocoded residential address and a measure of both weight and length or height at the same measurement visit (taken at the same time as or after the address date) from birth through adolescence (age range, 0-20 years). Of 69 ECHO cohorts, we included data from 54 cohorts participating in the ECHO program from January 1, 1995, to January 1, 2022 (eFigure 1 in Supplement 1), including 20 677 children who met the inclusion criteria and had available data within the data platform at the time of analyses. Data were analyzed from February 1 to June 30, 2022. Parents or guardians provided written informed consent for participation in the cohort of origin, and institutional review boards at each study site approved each local protocol. This study followed the Strengthening the Reporting of Observational Studies in Epidemiology (STROBE) reporting guideline for cohort studies.

### Exposure: Neighborhood Indices

Using ArcGIS geospatial software (Esri), we geocoded each participant’s residential address obtained at birth (year of residence from 1995-2022), infancy (median [IQR] age, 1.4 [1.3-1.5] years from 1996-2022), early childhood (median [IQR] age, 4.7 [3.3-4.8] years from 1998-2022), or mid-childhood (median [IQR] age, 8.2 [6.2-9.7] years from 2001-2022) and assigned a Census tract location to each participant address using the 1990, 2000, 2010, or 2020 US Census tract boundaries. At each life stage, we linked the Census tract location closest in time to the year of residence to the Census tract–level COI and SVI at the closest reference years for which data were available (ie, 2010 and 2015 for the COI; 2000, 2010, 2014, 2016, and 2018 for the SVI). For example, for children whose year of residence at birth was 2000, we linked the year 2000 Census tract location to the COI at year 2010 and the SVI at year 2000. Because COI measures were available in 2010 and 2015 only, COI data may have been misclassified for residential addresses during the 1990s or 2000s. This limitation was outweighed by the fact that a substantive majority of residential addresses (approximately 70%) at each life stage were obtained during or after the year 2010. Of 20 677 children included, 18 044 had both COI and SVI measures at birth, 17 300 had both measures in infancy (age range, 0.5-1.5 years), 15 452 had both measures in early childhood (age range, 2.0-4.8 years), and 11 190 had both measures in mid-childhood (age range, 5.0-9.8 years).

Researchers at Brandeis University developed the COI as a summary measure of the quality of neighborhoods in which children live across the US.^10^ This index quantifies 29 indicators of neighborhood conditions (eTable 1 in Supplement 1) that impact children’s healthy development, drawn from public sources and grouped into 3 domains: education, health and environment, and social and economic. The developers calculated domain-specific and overall COI scores for a total of 72 195 Census tracts across 50 US states and the District of Columbia. The scores were standardized at the national level such that higher scores reflected more favorable neighborhood opportunities relative to other neighborhoods across the US. The researchers also generated percentile ranks for each Census tract based on domain-specific and overall COI scores, ranging from the 1st percentile (lowest opportunity) to the 100th percentile (highest opportunity). In accordance with previous literature,^10,12^ we further grouped Census tract rankings into 5 categories: very low (<20th percentile), low (20th percentile to <40th percentile), moderate (40th percentile to <60th percentile), high (60th percentile to <80th percentile), or very high (≥80th percentile) opportunity.

The Centers for Disease Control and Prevention (CDC) developed and validated the SVI to identify high-risk populations that are especially vulnerable in the presence of a stressor or during public health emergencies.^11^ This index is derived from 15 US Census variables (eTable 2 in Supplement 1) grouped into 4 domains: socioeconomic status, household composition and disability, racial and ethnic minority and language status, and housing and transportation type. For each Census tract, the CDC generated percentile ranks, ranging from 0 (lowest vulnerability) to 1 (highest vulnerability) for all 15 variables collectively (ie, overall SVI) and for each of the 4 domains relative to all tracts across 50 states and the District of Columbia. To allow for direct comparison with COI measures, we also grouped these Census tract rankings for the SVI into very low (<20th percentile), low (20th percentile to <40th percentile), moderate (40th percentile to <60th percentile), high (60th percentile to <80th percentile), or very high (≥80th percentile) vulnerability.

### Outcomes: Childhood Body Mass Index and Obesity

Each participating cohort obtained at least 1 measure of weight and recumbent length (if aged <2 years) or standing height (if aged ≥2 years) in children at birth and in infancy, early childhood, mid-childhood, and/or adolescence based on their own established protocols. Sources of these anthropometric measurements included research visits, pediatric medical records, and mother or caregiver reports. Specific procedures and instruments used varied across the cohorts. We calculated BMI as weight in kilograms divided by length (if aged <2 years) or height in meters squared; the mean (SD) number of BMI measures per child was 5.0 (4.5). We excluded biologically implausible values if the BMI *z* score was less than 5 or greater than 5 for children younger than 2 years^20^ or less than 4 or greater than 8 for children 2 years and older.^21^ We defined obesity as having age- and sex-specific BMI greater than or equal to the 95th percentile according to the World Health Organization growth standards^20^ (if aged <2 years) or the CDC growth reference^21^ (if aged ≥2 years).

### Covariates

We obtained information on the following characteristics of mothers and children from maternal or caregiver reports or medical records: maternal educational level during pregnancy (less than high school, high school diploma or equivalent, some college but no degree, or college degree and higher), annual household income during pregnancy (<$50 000 or ≥$50 000 per year), prepregnancy BMI, total gestational weight gain, prenatal cigarette smoking, child’s sex (male or female), child’s race (American Indian or Alaska Native, Asian, Black, Native Hawaiian or Pacific Islander, White, multiple races, or other race), Hispanic ethnicity (yes or no), and year of child’s birth (before 2000, 2000-2010, or after 2010). Due to the small sample size, we combined children whose races were reported as American Indian or Alaska Native or as Native Hawaiian or Pacific Islander into a single category and children of multiple races or other race into another separate category. We viewed race and ethnicity as societal constructs rather than deterministic biological causes of disease risk^22^ and included information about race and ethnicity because we considered those characteristics as proxy measures of structural racism that can have implications for both residence in high-opportunity or low-vulnerability neighborhoods and access to resources that promote healthy weight.^23^ We also obtained information on pregnancy and birth characteristics including parity (nulliparous or multiparous), gestational diabetes (present or absent), gestational hypertension or preeclampsia (present or absent), mode of delivery (vaginal or cesarean), gestational age at delivery (in weeks), and birth weight (in kilograms) from medical records. We linked participants’ Census tract locations at each life stage to Census tract–level measures of rurality (metropolitan, micropolitan, or small town or rural areas) using Rural Urban Commuting Area codes from the US Department of Agriculture. We selected these covariates based on previous publications reporting an association between neighborhood environments and childhood obesity.^3,4,5,6,7^

### Statistical Analysis

We used linear mixed-effects models to examine associations of each neighborhood index (ie, COI or SVI) with children’s BMI over time. Details of these models are described in the eMethods in Supplement 1. We fitted separate models for each neighborhood index within each life stage; for each model, we included the following variables as fixed effects: neighborhood index categories, natural cubic spline terms for child’s age to capture the nonlinear trend in BMI across age,^24,25^ interactions of neighborhood index categories with each spline term for child’s age to capture the extent to which the association of neighborhood index with BMI may change across age, sociodemographic characteristics (ie, child’s sex, race, Hispanic or non-Hispanic ethnicity, and birth year and mother’s educational level and annual household income during pregnancy), and prenatal characteristics (ie, parity, gestational diabetes, gestational hypertension or preeclampsia, cigarette smoking, prepregnancy BMI, total gestational weight gain, mode of delivery, birth weight [only for models that included neighborhood index in infancy, early childhood, or mid-childhood], and gestational age at delivery). We included random effects for the intercept and linear slopes for child’s age to account for repeated BMI measures in the same child. We also included random effects for the cohort to account for clustering of children from the same cohort and random effects for the Census tract to account for clustering of children residing within the same neighborhood.

To ensure the exposure preceded the outcome, we fitted models including only BMI measures taken after the residential address date at each life stage (eg, when examining the association for COI linked to residential address in early childhood, we excluded BMI at birth and in infancy). We used these fitted models to estimate mean BMI over time for each neighborhood index category and plotted the corresponding BMI trajectory, holding all other covariates constant at their mean values. We estimated adjusted differences and 95% CIs for estimated BMI during infancy (age 0.5 years), early childhood (age 2 years), mid-childhood (ages 5 years and 10 years), and adolescence (ages 15 years and 20 years) in each COI and SVI category compared with the reference neighborhood index category (ie, very low COI or very high SVI).

To estimate the association of categories of neighborhood indices with the risk of childhood obesity (vs children without obesity) over time, we repeated all analyses using generalized linear mixed-effects models with a logit link, holding all covariates constant at their mean values. We used these models to estimate probabilities of obesity over time for each neighborhood index category, and we obtained risk ratios (RRs) and 95% CIs by taking the ratio of the marginal probabilities^26^ of obesity for each neighborhood index category to the reference neighborhood index category.

To compare COI and SVI at each life stage as factors associated with BMI and obesity, we used the overall *F* statistic from models estimating these outcomes.^27,28^ Given that at each life stage, the same number of children had measurements available from both neighborhood indices and each model was adjusted for the same set of covariates, we considered the neighborhood index in models with larger overall *F* statistics as the better estimator of BMI or obesity. We used a threshold of 5% or higher for the difference in *F* statistic values to indicate a meaningful advantage for the model with the larger value.^27,28^

We used multiple imputation by chained equations^29^ to impute values for missing covariate data (range, 1.7% to 53.1%) only (Table). We generated 50 imputed data sets for all 20 677 children who met the inclusion criteria. The imputation model included the exposure, outcome, and covariates under study. To impute missing data, we used estimated mean matching for continuous covariates, binary logistic regression models for dichotomous covariates, polytomous logistic regression models for unordered categorical covariates, and proportional odds methods for ordered categorical covariates. We combined the imputed data sets using the pool function in R software, version 4.1.0 (R Foundation for Statistical Computing).

**Table.** Participant Characteristics

Characteristic	Participants with nonmissing covariate data, No./total No. (%) (N = 20 677)	Imputed values for missing covariate data, %^a^
Education level during pregnancy^b^		
Less than high school	1024/12 627 (8.1)	8.7
High school diploma or equivalent	2077/12 627 (16.4)	17.2
Some college but no degree	2785/12 627 (22.1)	23.4
College degree and higher	6741/12 627 (53.4)	50.6
Annual household income during pregnancy, $^c^		
<50 000	4324/9690 (44.6)	47.4
≥50 000	5366/9690 (55.4)	52.6
Cigarette smoking during pregnancy^d^		
No	13 829/15 112 (91.5)	90.3
Yes	1283/15 112 (8.5)	9.7
Parity^e^		
Nulliparous	6015/15 382 (39.1)	38.9
Multiparous	9367/15 382 (60.9)	61.1
Gestational diabetes^f^		
No	13 611/15 102 (90.1)	90.5
Yes	1491/15 102 (9.9)	9.5
Gestational hypertension or preeclampsia^g^		
No	16 090/17 330 (92.8)	92.8
Yes	1240/17 330 (7.2)	7.2
Mode of delivery^h^		
Vaginal	11 419/16 841 (67.8)	68.2
Cesarean	5422/16 841 (32.2)	31.8
Prepregnancy BMI, mean (SD)^i^	26.8 (6.8)	26.8 (6.8)
Total gestational weight gain, mean (SD), kg^j^	15.1 (11.1)	15.0 (11.0)
Gestational age at delivery, mean (SD), wk^k^	37.8 (3.9)	37.8 (3.9)
Child’s birth year^l^		
Before 2000	221/19 624 (1.1)	1.1
2000-2010	5550/19 624 (28.3)	28.0
After 2010	13 853/19 624 (70.6)	70.9
Child’s sex		
Female	9930/20 677 (48.0)	NA
Male	10 747/20 677 (52.0)	NA
Child’s race^m^		
American Indian or Alaska Native and Native Hawaiian or Pacific Islander	454/20 105 (2.3)	2.3
Asian	684/20 105 (3.4)	3.4
Black	3047/20 105 (15.2)	15.1
White	12 463/20 105 (62.0)	61.6
Other race or >1 race	3457/20 105 (17.2)	17.6
Hispanic ethnicity^n^		
No	16 036/20 333 (78.9)	78.9
Yes	4297/20 333 (21.1)	21.1
No. of BMI measurements per child, mean (SD)	5.0 (4.5)	NA

Abbreviations: BMI, body mass index (calculated as weight in kilograms divided by length [if aged <2 years] or height in meters squared); NA, not applicable due to no missing covariate data.

^a^ Imputed characteristics are only shown as a percentage or mean (SD) of the 50 imputed data sets.

^b^ Data missing for 8050 participants (38.9%).

^c^ Data missing for 10 987 participants (53.1%).

^d^ Data missing for 5565 participants (26.9%).

^e^ Data missing for 5295 participants (25.6%).

^f^ Data missing for 5575 participants (27.0%).

^g^ Data missing for 3347 participants (16.2%).

^h^ Data missing for 3836 participants (18.6%).

^i^ Data missing for 4434 participants (21.4%).

^j^ Data missing for 7094 participants (34.3%).

^k^ Data missing for 1667 participants (8.1%).

^l^ Data missing for 1053 participants (5.1%).

^m^ Data missing for 572 participants (2.8%).

^n^ Data missing for 344 participants (1.7%).

We conducted several secondary analyses. First, in all models, we additionally adjusted for neighborhood index in all previous life stages but not in subsequent life stages (eg, adjusting COI in early childhood for previous COI measures at birth and infancy) to account for potential confounding by neighborhood indices during previous life stages. Second, we repeated all analyses using domain-specific COI or SVI categories to gain insights into specific facets of each that may have been associated with children’s BMI growth. Third, we restricted our analyses to residential addresses obtained during or after the year 2010 to address potential COI misclassification because COI was calculated for 2010. We explored effect modification by rurality of residence, child’s race and Hispanic or non-Hispanic ethnicity, child’s sex, and annual household income during pregnancy by stratifying all analyses according to these variables. We performed all analyses using R software, version 4.1.0. When interpreting findings, we focused primarily on the direction, strength, and precision of the estimates and used 2-sided α = .05 for assessment of statistical significance.

## Results

### Sample Characteristics

Among 20 677 children, 10 747 (52.0%) were male; 12 463 of 20 105 (62.0%) were White, and 16 036 of 20 333 (78.9%) were non-Hispanic (some data for race and ethnicity were missing) (Table). Overall, 29.9% of children in the ECHO program resided in areas with the most advantageous characteristics. Based on nationwide distributions of the COI, 20.8% of children at birth, 20.0% of children in infancy, 18.9% of children in early childhood, and 16.9% of children in mid-childhood resided in areas with very low overall COI, while 26.7% of children at birth, 27.8% of children in infancy, 29.4% of children in early childhood, and 30.6% of children in mid-childhood resided in areas with very high overall COI (eTable 3 in Supplement 1). The proportions of children who resided in areas with very high or very low overall SVI at each life stage were similar to those of the COI (eTable 4 in Supplement 1). A total of 25.3% of children at birth, 26.0% of children in infancy, 26.8% of children in early childhood, and 28.4% of children in mid-childhood lived in areas with very low overall SVI, whereas 21.1% of children at birth, 20.5% of children in infancy, 19.3% of children in early childhood, and 16.9% of children in mid-childhood lived in areas with very high overall SVI. Because the expected national distribution was approximately 20% within each category, this study population was generally representative of the exposure distribution of US children. During every life stage, we noted substantial negative correlations between the 2 indices (birth: ρ = –0.86; infancy: ρ = –0.87; early childhood: ρ = –0.88; mid-childhood: ρ = –0.87), which was expected given that the COI represents a compilation of positive opportunities, while the SVI represents a compilation of adverse characteristics. The characteristics of the study sample according to COI and SVI categories are shown in eTable 5 and eTable 6 in Supplement 1, respectively.

### Association of COI With Childhood BMI and Obesity

**Figure 1.**
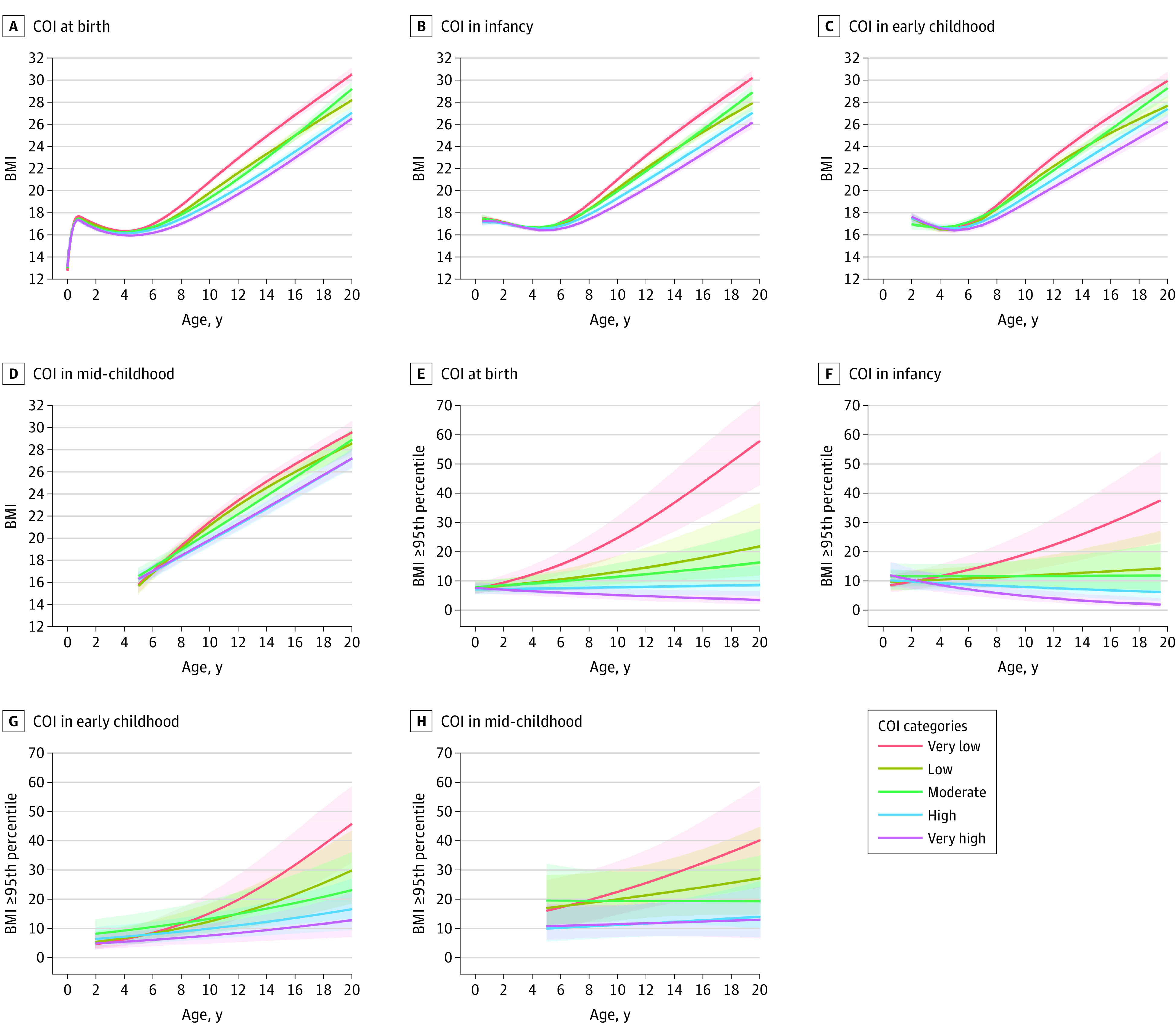
Trajectories of Body Mass Index (BMI) and Probability of Obesity From Birth to Adolescence According to Child Opportunity Index (COI) Categories Adjusted for sociodemographic and prenatal characteristics. Shaded regions represent 95% CIs. BMI was calculated as weight in kilograms divided by length (if aged <2 years) or height in meters squared.

**Figure 2.**
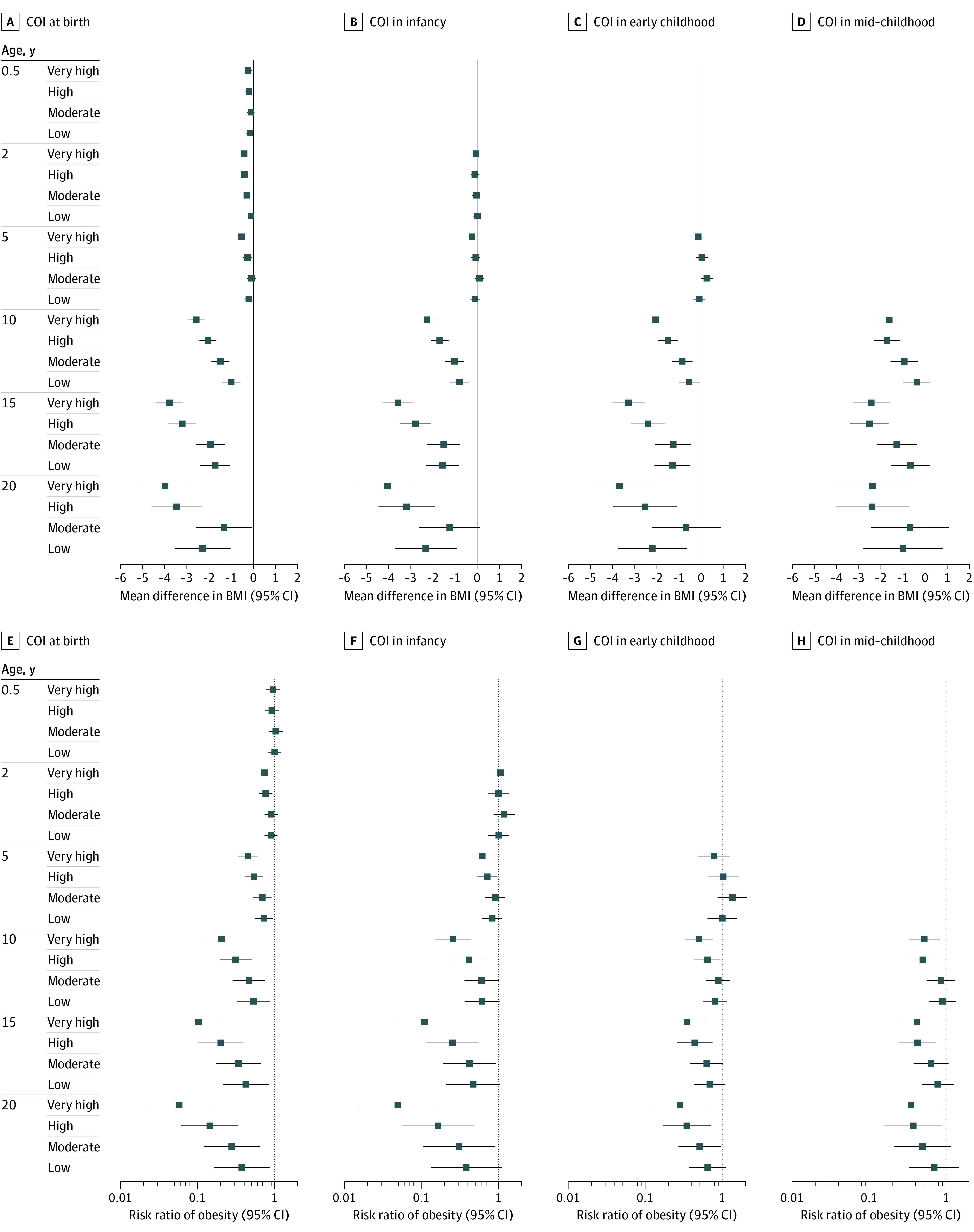
Association of Child Opportunity Index (COI) Categories at Different Life Stages With Mean Difference in Body Mass Index (BMI) and Risk of Obesity All effect estimates and 95% CIs are relative to the very low COI category and adjusted for sociodemographic and prenatal characteristics. BMI was calculated as weight in kilograms divided by length (if aged <2 years) or height in meters squared.

After adjusting for family sociodemographic and prenatal characteristics, linear mixed-effects models revealed dose-response associations at every life stage, wherein children who resided in areas with higher COI had lower mean subsequent BMI (Figure 1A-D) and lower probability of obesity over time (Figure 1E-H) compared with those who resided in areas with very low COI. For example, effect estimates for mean BMI difference (β = –2.58; 95% CI, –2.95 to –2.21) and risk of obesity (RR, 0.21; 95% CI, 0.12-0.34) at age 10 years were larger among children who resided in areas with very high (vs very low) COI at birth, with a smaller (but still significant) mean BMI difference and risk of obesity among those who resided in areas with high COI (BMI: β = –2.05 [95% CI, –2.43 to –1.67]; obesity: RR, 0.31 [95% CI, 0.20-0.51]), moderate COI (BMI: β = –1.51 [95% CI, –1.91 to –1.10]; obesity: RR, 0.46 [95% CI, 0.28-0.74]), and low COI (BMI: β = –1.01 [95% CI, –1.43 to –0.60]; obesity: RR, 0.53 [95% CI, 0.32-0.86]) (Figure 2).

Furthermore, effect estimates for mean BMI difference and obesity risk were larger at an older age of outcome measurement. Specifically, effect estimates for the mean BMI difference in children who resided in areas with very high (vs very low) COI at birth were −0.52 (95% CI, –0.72 to –0.33) at 5 years, –2.58 (95% CI, –2.95 to –2.21) at 10 years, and –3.78 (95% CI, –4.40 to –3.17) at 15 years, while the risks of obesity were 0.45 (95% CI, 0.34-0.59) at 5 years, 0.21 (95% CI, 0.12-0.34) at 10 years, and 0.10 (95% CI, 0.05-0.20) at 15 years. We noted similar observations at other life stages (Figure 2).

Overall, effect estimates for mean BMI difference and risk of obesity were larger for COI exposure at birth compared with COI exposure at later life stages. For example, children who resided in areas with very high (vs very low) COI at birth had the most substantial mean BMI difference (β = –2.58; 95% CI, –2.95 to –2.21) and risk of obesity (RR, 0.21; 95% CI, 0.12-0.34) at age 10 years relative to those who resided in areas with very high (vs very low) COI in infancy (BMI: β = –2.26 [95% CI, –2.65 to –1.87]; obesity: RR, 0.26 [95% CI, 0.15-0.44]), early childhood (BMI: β = –2.05 [95% CI, –2.47 to –1.66]; obesity: RR, 0.50 [95% CI, 0.33-0.75), or mid-childhood (BMI: β = –1.61 [95% CI, –2.21 to –1.02]; obesity: RR, 0.52 [95% CI, 0.33-0.83]) (Figure 2).

### Association of SVI With Childhood BMI and Obesity

**Figure 3.**
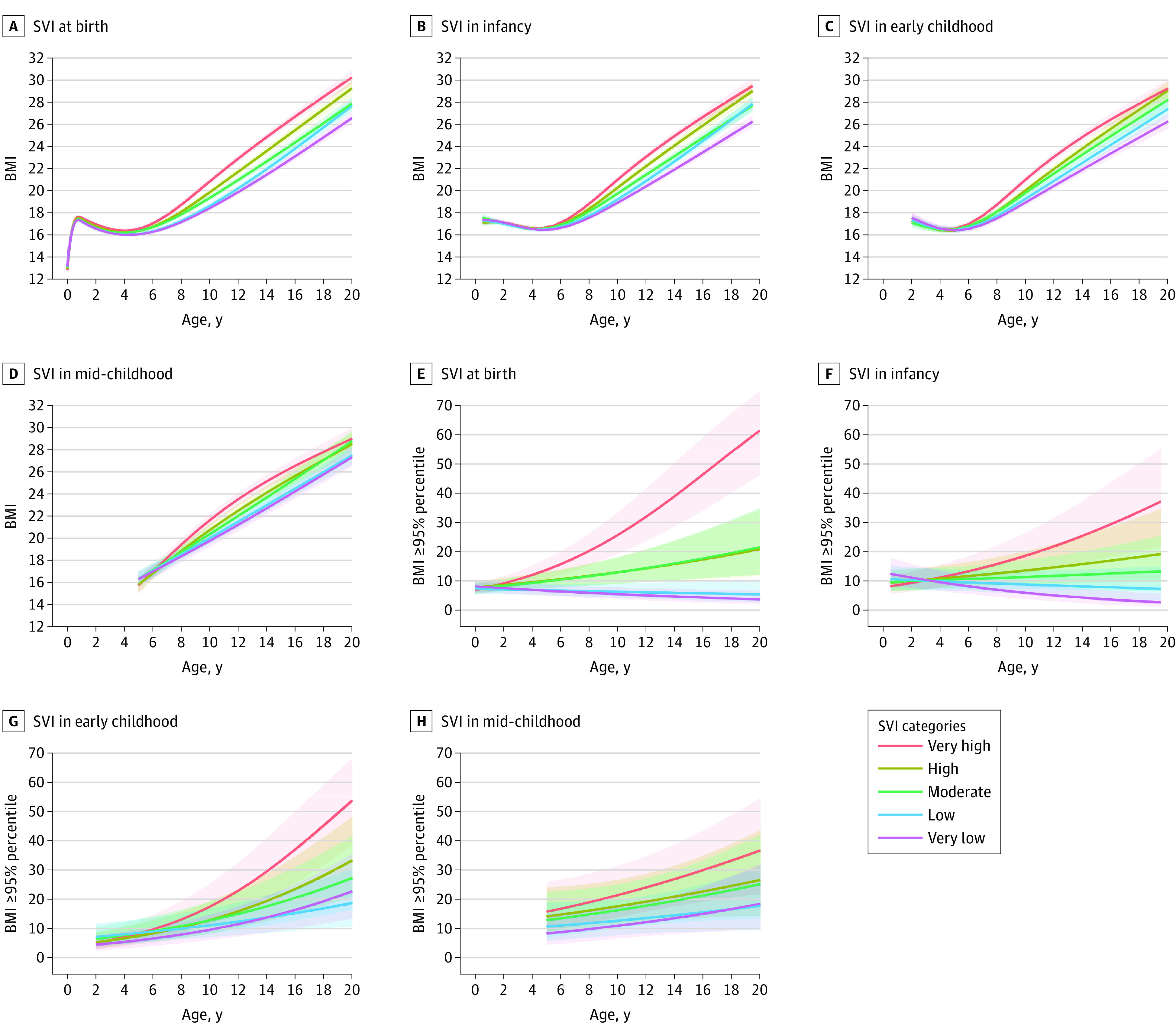
Trajectories of Body Mass Index (BMI) and Probability of Obesity From Birth to Adolescence According to Social Vulnerability Index (SVI) Categories Adjusted for sociodemographic and prenatal characteristics. Shaded regions represent 95% CIs. BMI was calculated as weight in kilograms divided by length (if aged <2 years) or height in meters squared.

**Figure 4.**
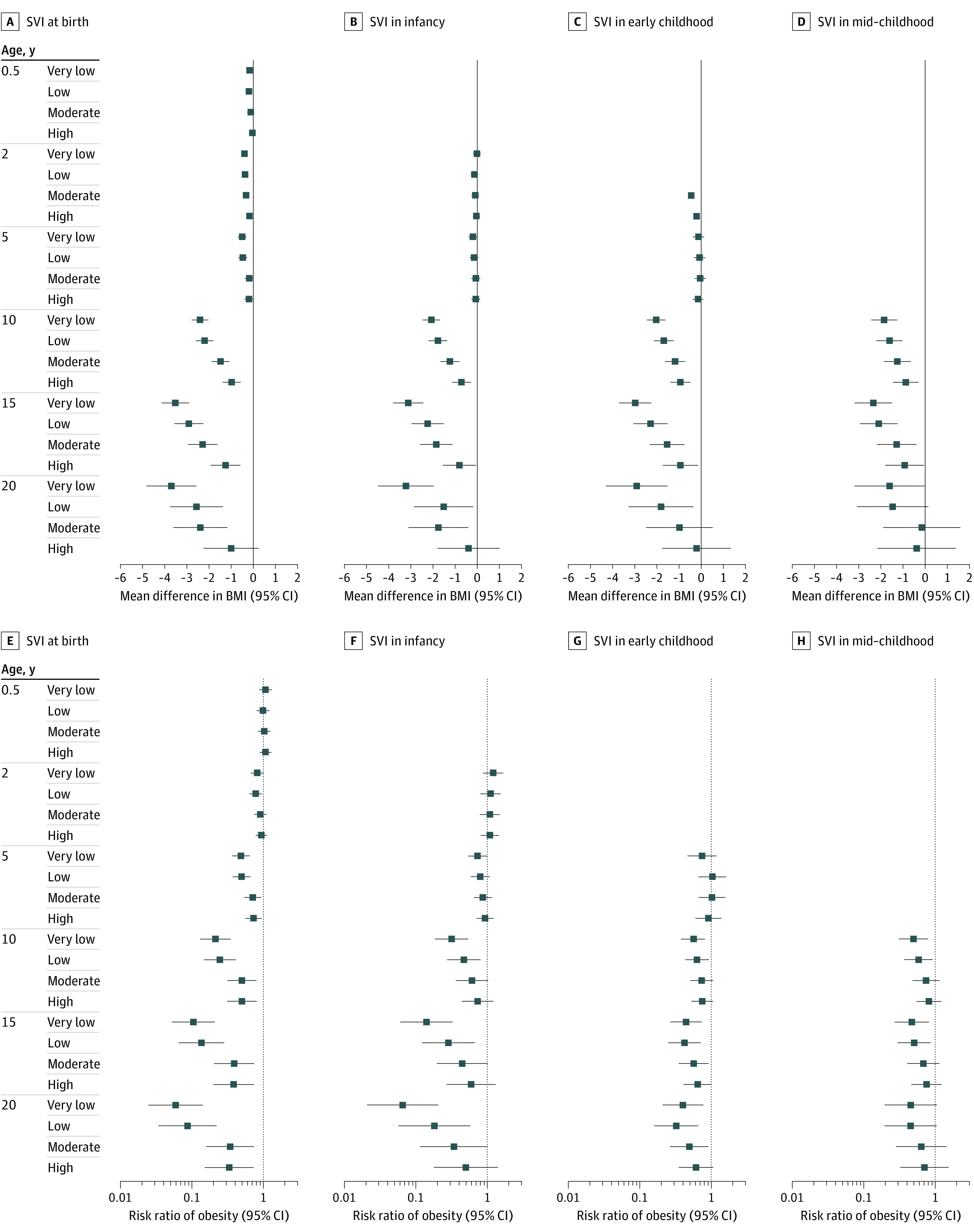
Association of Social Vulnerability Index (SVI) Categories at Different Life Stages With Mean Difference in Body Mass Index (BMI) and Risk of Obesity All effect estimates and 95% CIs are relative to the very high SVI category and adjusted for sociodemographic and prenatal characteristics. BMI was calculated as weight in kilograms divided by length (if aged <2 years) or height in meters squared.

Similar observations were noted for the association of SVI with childhood BMI and obesity. First, we noted dose-response associations at every life stage, wherein children who resided in areas with lower SVI had significantly lower mean subsequent BMI (Figure 3A-D) and lower probability of obesity (Figure 3E-H) over time compared with those who resided in areas with very high SVI. For example, effect estimates for mean BMI difference (β = –2.40; 95% CI, –2.78 to –2.03) and risk of obesity (RR, 0.17; 95% CI, 0.10-0.30) at age 10 years were larger among children who resided in areas with very low (vs very high) SVI at birth, with a smaller (but still significant) mean BMI difference and risk of obesity among those who resided in areas with low SVI (BMI: β = –2.20 [95% CI, –2.60 to –1.81]; obesity: RR, 0.20 [95% CI, 0.11-0.35]), moderate SVI (BMI: β = –1.48 [95% CI, –1.88 to –1.08]; obesity: RR, 0.42 [95% CI, 0.24-0.75]), and high SVI (BMI: β = –0.98 [95% CI, –1.39 to –0.58]; obesity: RR, 0.43 [95% CI, 0.24-0.76]). Second, effect estimates for mean BMI difference and risk of obesity were larger at an older age of outcome measurement. For instance, effect estimates for the mean BMI difference in children who resided in areas with very low (vs very high) SVI at birth were −0.50 (95% CI, –0.69 to –0.31) at 5 years, –2.41 (95% CI, –2.78 to –2.03) at 10 years, and –3.52 (95% CI, –4.13 to –2.90) at 15 years, while the risks of obesity were 0.45 (95% CI, 0.33-0.61) at 5 years, 0.17 (95% CI, 0.10-0.30) at 10 years, and 0.08 (95% CI, 0.03-0.20) at 15 years. Third, the mean BMI difference and risk of obesity was greater for SVI exposure at birth compared with SVI exposure at later life stages (Figure 4). Based on *F* statistics, neither neighborhood index at each life stage was superior to the other in its association with childhood BMI and obesity (eg, at birth: *F* = 17452.39 for the COI and 17416.19 for the SVI) (eTable 7 in Supplement 1).

### Secondary Analyses

We noted no appreciable changes to the effect estimates after additional adjustment for COI or SVI at previous life stages (eFigure 2 and eFigure 3 in Supplement 1). Additionally, for each domain of the COI or SVI, the associations with BMI and risk of obesity were largely similar in significance and direction to the overall index (eFigure 4 and eFigure 5 in Supplement 1). We noted similar associations for COI when restricting the analyses to residential addresses obtained during or after the year 2010 (eFigure 6 in Supplement 1). Additionally, exposure to COI or SVI was associated with larger differences in subsequent mean BMI and risk of obesity in Hispanic children (eFigure 7 and eFigure 8 in Supplement 1). For example, Hispanic children who resided in areas with very high (vs very low) COI at birth had larger mean BMI differences (β = –3.13; 95% CI, –4.31 to –1.94) and lower risk of obesity (RR, 0.11; 95% CI, 0.02-0.59) at age 10 years compared with non-Hispanic White children (BMI: β –1.01 [95% CI, –1.64 to –0.38]; obesity: RR, 0.52 [95% CI, 0.25-1.07]) and non-Hispanic Black children (BMI: β = –0.28 [95% CI, –1.96 to 1.40]; obesity: RR, 0.42 [95% CI, 0.06-2.83]) residing in the same area. However, there was no clear evidence of effect modification by rurality of residence, child’s sex, or household income during pregnancy.

## Discussion

In this nationwide cohort study, we found that US children who resided in higher-opportunity or less vulnerable neighborhoods had a lower mean BMI trajectory and a lower risk of obesity from childhood to adolescence. Effect estimates for mean BMI difference and risk of obesity were larger for children residing in neighborhoods with the highest opportunity or lowest vulnerability. These associations were independent of individual and family sociodemographic factors as well as prenatal factors that are established risk factors for childhood obesity.^30,31,32,33^ Notably, exposure to neighborhood-level opportunity or vulnerability measures at birth was associated with the most substantial difference in subsequent mean BMI and risk of obesity compared with exposure at later life stages.

Our findings are consistent with those of previous studies,^34,35,36^ which reported that exposure to neighborhoods with higher disadvantage was associated with higher BMI and increased risk of obesity across childhood. Previous studies, however, have often been limited by small samples (approximately 500 participants),^36^ lack of geographical diversity,^34,35^ and insufficient variation in individual-level characteristics,^3,37^ all of which might have hampered the ability to detect these associations. Moreover, most existing work on the association between neighborhoods and childhood BMI did not examine neighborhood characteristics in very early life or were limited to cross-sectional designs that omitted the temporal nature of these associations.^38,39^ Furthermore, longitudinal studies^34,35^ that examined associations of neighborhood environments with childhood BMI or obesity have used indices that represent only specific aspects of socioeconomic disadvantage, such as unemployment and low household income. We directly addressed these key research gaps by (1) leveraging the ECHO infrastructure to assemble a geographically diverse cohort (eFigure 1 in Supplement 1) of more than 20 000 children with repeated residential and anthropometric data, making this study, to our knowledge, the largest to examine the association of neighborhood indices with BMI changes from birth through adolescence, and (2) examining novel neighborhood indices (ie, the COI) that incorporate both positive and negative attributes of neighborhood conditions that may promote or inhibit healthy development in children.

It is worth noting that exposure to the highest opportunity or least vulnerable neighborhoods was associated with substantial mean differences in BMI (by approximately 2 kg/m^2^) and obesity risk (by approximately 80%) in childhood and adolescence. The clinical benefits of reducing childhood obesity are well known. For example, a previous study^40^ among children aged 5 to 17 years with overweight reported that a reduction of BMI *z* score by 0.25 units (approximately 1 kg/m^2^) was associated with significant decreases in blood pressure, triglyceride levels, and insulin resistance, all of which are clinical markers of cardiovascular health,^41^ 1 year later. Additionally, a simulation study^42^ using data from the National Medical Expenditure Panel Survey reported that a 1% reduction in the prevalence of overweight and obesity in adolescents would not only reduce the prevalence of obesity in adulthood but also substantially increase quality-adjusted life years after age 40 years. Taken together, our findings support the notion that residence in a high-opportunity or low-vulnerability neighborhood might constitute an important resilience factor that may promote development of favorable BMI patterns that, in turn, could potentially mitigate future chronic disease risk.

We found that residence in higher-opportunity or lower-vulnerability neighborhoods was associated with significantly lower mean BMI trajectories and significantly lower risk of obesity in children when outcomes were assessed at older ages. We speculate that this phenomenon may partly be explained by cumulative exposure to resource deprivation or benefit over time that may operate jointly to improve child outcomes.^43^

Researchers have proposed that childhood compared with earlier life stages may be a particularly sensitive period for exposure to neighborhood environments because this life stage reflects a developmental window when health behaviors (eg, dietary habits and physical activity) may be established.^44^ However, we found that exposure to COI or SVI at birth was associated with the most substantial difference in mean BMI and risk of obesity compared with exposure at later life stages. We are aware of only 1 other study that has found similar associations; Jimenez et al^45^ reported that neighborhood socioeconomic status at birth rather than in childhood or adulthood was associated with systolic and diastolic blood pressure in adulthood. One possible explanation is that residence in neighborhoods with the highest opportunity or lowest vulnerability at birth likely reflects improved access to essential resources such as healthy food choices during pregnancy, a critical developmental period for the fetus,^46^ which may have strong, healthful, and long-lasting implications for children’s growth. We did not, however, evaluate associations between neighborhood indices and specific health behaviors in children. Future research may consider evaluating how efforts to improve neighborhood environment could alter social and behavioral factors associated with child health outcomes, such as social support and physical activity.^47,48^

We noted that the COI and SVI exhibited comparable associations with childhood BMI and obesity, suggesting that either index could be useful screening tools to identify vulnerable children at high risk of progressing to a trajectory of high BMI. While the SVI has more reference years available, the COI may be preferable because it was developed specifically for children’s health and incorporates more novel attributes of neighborhood conditions. Nevertheless, both the COI and SVI could be used to target the development and potential surveillance of obesity-related outcomes associated with place-based initiatives, strategies, or policies that directly address the disparate contexts of neighborhoods, reduce barriers and improve access to essential resources, and provide families with the environments needed to support optimal childhood health and well-being.

Our findings of an association between exposure to neighborhood-level opportunity or vulnerability measures and larger differences in mean BMI and risk of obesity in Hispanic children is consistent with a previous study that reported Hispanic children who lived in high-opportunity neighborhoods (vs those who did not) were more physically active.^49^ It has been speculated that residence in a high-opportunity neighborhood may increase exposure to visual cues of other children actively using resources in the neighborhood, which may subsequently alter children’s behavior and health outcomes.^49^ This observation was previously found to be more impactful for Hispanic individuals compared with non-Hispanic White individuals.^50,51^ Given that Hispanic children are known to be at increased risk of obesity compared with non-Hispanic White children,^52^ further studies are warranted to investigate the mechanisms behind these different associations.

### Strengths and Limitations

This study has several strengths. Other than its large sample, strengths include the study’s long-term follow-up and wide range of covariates. Furthermore, children in the study sample resided in diverse geographic regions across the US, which not only makes this study nationally representative but also improves the generalizability of its results. We used neighborhood indices that captured both physical (eg, access to green space) and social (eg, access to health care services) attributes, and the indices have been validated for a range of health outcomes.^12,13^ We assessed neighborhood opportunity and vulnerability at life stages during which children were unlikely to select their place of residence, which reduces the likelihood of self-selection and potential reverse causation bias (ie, BMI having consequences for the place of residence). We also controlled for maternal prepregnancy BMI, which reduces the likelihood of residential self-selection by parental factors that might be associated with childhood BMI.

The study also has limitations. First, we lacked direct measures of children’s adiposity and instead used BMI, which is an imperfect measure of adiposity. Thus, our results may not reflect the true association between neighborhood environments and children’s adiposity. However, obesity is more directly associated with excess adiposity,^53^ and our results for the risk of obesity paralleled those for BMI. Second, we used residential Census tracts as a marker of exposure, which may not capture the relevant areas where children spend all of their time. Third, the COI and SVI comprise many individual indicators that are correlated with each other, making it difficult to distinguish which component of the neighborhood is the most important factor in the association with BMI and obesity. Fourth, both indices were limited to components for which nationally representative data were available. Other neighborhood-level social attributes that might have implications for children’s health, such as exposure to neighborhood violence or poor social support or cohesion,^54,55^ were not included because of a lack of comparable data across the US. Fifth, COI information is available for 2010 and 2015 only and may be misclassified for residential addresses during the 1990s or 2000s. However, results for COI restricted to residential addresses obtained during or after the year 2010 were similar to our main findings. Sixth, we did not examine the extent to which neighborhood mobility from birth to mid-childhood was associated with children’s BMI, an important question given that previous research has revealed the long-term socioeconomic benefits (ie, increased college attendance and adult earnings) of moving from lower to higher-opportunity neighborhoods early in life.^56^ While this question is beyond the scope of the current study, follow-up studies of ECHO cohorts to investigate these associations throughout childhood are possible and will be helpful to develop more beneficial policy strategies, especially given demonstrated interest by policy makers in addressing obesogenic environments in the US.^57^

## Conclusions

The results of this cohort study suggest that residing in neighborhoods with higher-opportunity or lower-vulnerability early in life, especially at birth, is associated with lower mean BMI trajectory and lower risk of obesity from childhood to adolescence. In recognition of the long-term sequelae stemming from high BMI or obesity in childhood, this study’s findings support the need for a focus on investments that address the structures that consistently compromise the health of marginalized communities. More research is warranted to clarify whether initiatives or policies that alter specific components of neighborhood environment would be beneficial in preventing excess weight and obesity in children.

## Notes

Supplement 1. **eTable 1.** Child Opportunity Index Indicators, Definitions, and Data Sources **eTable 2.** Social Vulnerability Index Indicators, Definitions, and Data Sources **eTable 3.** Child Opportunity Index and Rurality of Residence at Each Life Stage **eTable 4.** Social Vulnerability Index at Each Life Stage **eTable 5.** Participant Characteristics According to Very Low and Very High Child Opportunity Index (ChOI) Categories at Each Life Stage **eTable 6.** Participant Characteristics According to Very Low and Very High Social Vulnerability Index (SVI) Categories at Each Life Stage **eTable 7.** Overall *F* Statistic Values for Child Opportunity Index (ChOI) and Social Vulnerability Index (SVI) Models at Each Life Stage **eFigure 1.** Recruitment Site Locations for Cohorts Included in the Analytic Sample **eFigure 2.** Association of Child Opportunity Index Categories at Different Life Stages With Mean Difference in Body Mass Index and Risk of Obesity at Ages 0.5, 2, 5, 10, 15, and 20 Years **eFigure 3.** Association of Social Vulnerability Index Categories at Different Life Stages With Mean Difference in Body Mass Index and Risk of Obesity at Ages 0.5, 2, 5, 10, 15, and 20 Years **eFigure 4.** Association of Domain-Specific Child Opportunity Index Categories at Different Life Stages With Mean Difference in Body Mass Index and Risk of Obesity at Ages 10 and 15 Years **eFigure 5.** Association of Domain-Specific Social Vulnerability Index Categories at Different Life Stages With Mean Difference in Body Mass Index and Risk of Obesity at Ages 10 and 15 Years **eFigure 6.** Association of Child Opportunity Index Categories at Different Life Stages With Mean Difference in Body Mass Index and Risk of Obesity at Ages 0.5, 2, 5, 10, 15, and 20 Years Restricted to Residential Addresses Obtained During or After Year 2010 **eFigure 7.** Association of Child Opportunity Index Categories at Different Life Stages With Mean Difference in Body Mass Index and Risk of Obesity at Ages 10 and 15 Years by Child’s Race and Hispanic Ethnicity **eFigure 8.** Association of Social Vulnerability Index Categories at Different Life Stages With Mean Difference in Body Mass Index and Risk of Obesity at Ages 10 and 15 Years by Child’s Race and Hispanic Ethnicity **eMethods.** Procedure for Estimating Body Mass Index Trajectories
eReferences

Supplement 2. **Nonauthor Collaborators.** The Program Collaborators for Environmental Influences on Child Health Outcomes

Supplement 3.
Data Sharing Statement

## References

[1] Sellström E, Bremberg S. The significance of neighbourhood context to child and adolescent health and well-being: a systematic review of multilevel studies. Scand J Public Health. 2006;34(5):544-554. doi:10.1080/14034940600551251 16990166

[2] Christian H, Zubrick SR, Foster S, . The influence of the neighborhood physical environment on early child health and development: a review and call for research. Health Place. 2015;33:25-36. doi:10.1016/j.healthplace.2015.01.005 25744220

[3] Singh GK, Siahpush M, Kogan MD. Neighborhood socioeconomic conditions, built environments, and childhood obesity. Health Aff (Millwood). 2010;29(3):503-512. doi:10.1377/hlthaff.2009.0730 20194993

[4] Gartstein MA, Seamon E, Thompson SF, Lengua LJ. Featured article: community crime exposure and risk for obesity in preschool children: moderation by the hypothalamic-pituitary-adrenal-axis response. J Pediatr Psychol. 2018;43(4):353-365. doi:10.1093/jpepsy/jsx116 29048574 PMC6927899

[5] Bjerregaard LG, Jensen BW, Ängquist L, Osler M, Sørensen TIA, Baker JL. Change in overweight from childhood to early adulthood and risk of type 2 diabetes. N Engl J Med. 2018;378(14):1302-1312. doi:10.1056/NEJMoa1713231 29617589

[6] Buka SL, Brennan RT, Rich-Edwards JW, Raudenbush SW, Earls F. Neighborhood support and the birth weight of urban infants. Am J Epidemiol. 2003;157(1):1-8. doi:10.1093/aje/kwf170 12505884

[7] Yang Y, Jiang Y, Xu Y, Mzayek F, Levy M. A cross-sectional study of the influence of neighborhood environment on childhood overweight and obesity: variation by age, gender, and environment characteristics. Prev Med. 2018;108:23-28. doi:10.1016/j.ypmed.2017.12.021 29289640

[8] Mohammed SH, Habtewold TD, Birhanu MM, . Neighbourhood socioeconomic status and overweight/obesity: a systematic review and meta-analysis of epidemiological studies. BMJ Open. 2019;9(11):e028238. doi:10.1136/bmjopen-2018-028238 31727643 PMC6886990

[9] Daelmans B, Darmstadt GL, Lombardi J, ; Lancet Early Childhood Development Series Steering Committee. Early childhood development: the foundation of sustainable development. Lancet. 2017;389(10064):9-11. doi:10.1016/S0140-6736(16)31659-2 27717607

[10] Acevedo-Garcia D, Noelke C, McArdle N, . Racial and ethnic inequities in children’s neighborhoods: evidence from the new Child Opportunity Index 2.0. Health Aff (Millwood). 2020;39(10):1693-1701. doi:10.1377/hlthaff.2020.00735 33017244

[11] Flanagan BE, Hallisey EJ, Adams E, Lavery A. Measuring community vulnerability to natural and anthropogenic hazards: the Centers for Disease Control and Prevention’s Social Vulnerability Index. J Environ Health. 2018;80(10):34-36. 32327766 PMC7179070

[12] Aris IM, Rifas-Shiman SL, Jimenez MP, . Neighborhood Child Opportunity Index and adolescent cardiometabolic risk. Pediatrics. 2021;147(2):e2020018903. doi:10.1542/peds.2020-018903 33479165 PMC7906069

[13] Bevan G, Pandey A, Griggs S, . Neighborhood-level social vulnerability and prevalence of cardiovascular risk factors and coronary heart disease. Curr Probl Cardiol. Published online March 27, 2022. doi:10.1016/j.cpcardiol.2022.101182 35354074 PMC9875801

[14] Yu CY, Woo A, Emrich CT, Wang B. Social Vulnerability Index and obesity: an empirical study in the US. *Cities*. 2020;97:102531. doi:10.1016/j.cities.2019.102531

[15] Aris IM, Bernard JY, Chen LW, . Postnatal height and adiposity gain, childhood blood pressure and prehypertension risk in an Asian birth cohort. Int J Obes (Lond). 2017;41(7):1011-1017. doi:10.1038/ijo.2017.40 28186098 PMC5473468

[16] Zhang X, Martin RM, Oken E, Aris IM, Yang S, Kramer MS. Growth during infancy and early childhood and its association with metabolic risk biomarkers at 11.5 years of age. Am J Epidemiol. 2020;189(4):286-293. doi:10.1093/aje/kwz234 31595955 PMC7305788

[17] Zhang X, Tilling K, Martin RM, . Analysis of ‘sensitive’ periods of fetal and child growth. Int J Epidemiol. 2019;48(1):116-123. doi:10.1093/ije/dyy045 29618044 PMC6380295

[18] LeWinn KZ, Caretta E, Davis A, Anderson AL, Oken E; Program Collaborators for Environmental Influences on Child Health Outcomes. SPR perspectives: Environmental Influences on Child Health Outcomes (ECHO) program: overcoming challenges to generate engaged, multidisciplinary science. Pediatr Res. Published online June 15, 2021. doi:10.1038/s41390-021-01598-0 PMC8204620 34131290

[19] Blaisdell CJ, Park C, Hanspal M, ; Program Collaborators for Environmental Influences on Child Health Outcomes. The NIH ECHO program: investigating how early environmental influences affect child health. Pediatr Res. Published online June 15, 2021. doi:10.1038/s41390-021-01574-8 34131291 PMC8204611

[20] de Onis M, Onyango AW, Borghi E, Siyam A, Nishida C, Siekmann J. Development of a WHO growth reference for school-aged children and adolescents. Bull World Health Organ. 2007;85(9):660-667. doi:10.2471/BLT.07.043497 18026621 PMC2636412

[21] Kuczmarski RJ, Ogden CL, Guo SS, . 2000 CDC growth charts for the United States: methods and development. Vital Health Stat 11. 2002;(246):1-190. 12043359

[22] Flanagin A, Frey T, Christiansen SL; AMA Manual of Style Committee. Updated guidance on the reporting of race and ethnicity in medical and science journals. JAMA. 2021;326(7):621-627. doi:10.1001/jama.2021.13304 34402850

[23] Bell CN, Kerr J, Young JL. Associations between obesity, obesogenic environments, and structural racism vary by county-level racial composition. Int J Environ Res Public Health. 2019;16(5):861. doi:10.3390/ijerph16050861 30857286 PMC6427384

[24] Aris IM, Rifas-Shiman SL, Li LJ, . Pre-, perinatal, and parental predictors of body mass index trajectory milestones. J Pediatr. 2018;201:69-77. doi:10.1016/j.jpeds.2018.05.041 29960766 PMC6153023

[25] Aris IM, Rifas-Shiman SL, Li LJ, . Patterns of body mass index milestones in early life and cardiometabolic risk in early adolescence. Int J Epidemiol. 2019;48(1):157-167. doi:10.1093/ije/dyy286 30624710 PMC6380298

[26] Austin PC. Absolute risk reductions, relative risks, relative risk reductions, and numbers needed to treat can be obtained from a logistic regression model. J Clin Epidemiol. 2010;63(1):2-6. doi:10.1016/j.jclinepi.2008.11.004 19230611

[27] Aris IM, Rifas-Shiman SL, Li LJ, . Association of weight for length vs body mass index during the first 2 years of life with cardiometabolic risk in early adolescence. JAMA Netw Open. 2018;1(5):e182460. doi:10.1001/jamanetworkopen.2018.2460 30646168 PMC6324504

[28] Kleinman KP, Oken E, Radesky JS, Rich-Edwards JW, Peterson KE, Gillman MW. How should gestational weight gain be assessed? a comparison of existing methods and a novel method, area under the weight gain curve. Int J Epidemiol. 2007;36(6):1275-1282. doi:10.1093/ije/dym156 17715174 PMC2157551

[29] Li P, Stuart EA, Allison DB. Multiple imputation: a flexible tool for handling missing data. JAMA. 2015;314(18):1966-1967. doi:10.1001/jama.2015.15281 26547468 PMC4638176

[30] Kuhle S, Tong OS, Woolcott CG. Association between caesarean section and childhood obesity: a systematic review and meta-analysis. Obes Rev. 2015;16(4):295-303. doi:10.1111/obr.12267 25752886

[31] Patro Golab B, Santos S, Voerman E, Lawlor DA, Jaddoe VWV, Gaillard R; MOCO Study Group Authors. Influence of maternal obesity on the association between common pregnancy complications and risk of childhood obesity: an individual participant data meta-analysis. Lancet Child Adolesc Health. 2018;2(11):812-821. doi:10.1016/S2352-4642(18)30273-6 30201470 PMC6196075

[32] Voerman E, Santos S, Patro Golab B, . Maternal body mass index, gestational weight gain, and the risk of overweight and obesity across childhood: an individual participant data meta-analysis. PLoS Med. 2019;16(2):e1002744. doi:10.1371/journal.pmed.1002744 30742624 PMC6370184

[33] Philips EM, Santos S, Trasande L, . Changes in parental smoking during pregnancy and risks of adverse birth outcomes and childhood overweight in Europe and North America: an individual participant data meta-analysis of 229,000 singleton births. PLoS Med. 2020;17(8):e1003182. doi:10.1371/journal.pmed.1003182 32810184 PMC7433860

[34] Alvarado SE. Neighborhood disadvantage and obesity across childhood and adolescence: evidence from the NLSY children and young adults cohort (1986-2010). Soc Sci Res. 2016;57:80-98. doi:10.1016/j.ssresearch.2016.01.008 26973033

[35] Rautava S, Turta O, Vahtera J, . Neighborhood socioeconomic disadvantage and childhood body mass index trajectories from birth to 7 years of age. Epidemiology. 2022;33(1):121-130. doi:10.1097/EDE.0000000000001420 34669629 PMC8614531

[36] Thorpe D, Klein DN. The effect of neighborhood-level resources on children’s physical development: trajectories of body mass index and pubertal development and the influence of child biological sex. J Youth Adolesc. 2022;51(5):967-983. doi:10.1007/s10964-021-01547-4 35028875

[37] Lovasi GS, Schwartz-Soicher O, Quinn JW, . Neighborhood safety and green space as predictors of obesity among preschool children from low-income families in New York City. Prev Med. 2013;57(3):189-193. doi:10.1016/j.ypmed.2013.05.012 23732240 PMC3748212

[38] Daniels KM, Lê-Scherban F, Schinasi LH, . Cross-sectional associations of built and social neighborhood environment variables with body mass index in a large sample of urban predominantly African American children. Child Obes. 2021;17(3):209-219. doi:10.1089/chi.2020.0155 33555978

[39] Paciência I, Cavaleiro Rufo J, Mendes F, . A cross-sectional study of the impact of school neighbourhood on children obesity and body composition. Eur J Pediatr. 2021;180(2):535-545. doi:10.1007/s00431-020-03798-y 32910211

[40] Reinehr T, Lass N, Toschke C, Rothermel J, Lanzinger S, Holl RW. Which amount of BMI-SDS reduction is necessary to improve cardiovascular risk factors in overweight children? J Clin Endocrinol Metab. 2016;101(8):3171-3179. doi:10.1210/jc.2016-1885 27285295

[41] Magnussen CG, Koskinen J, Chen W, . Pediatric metabolic syndrome predicts adulthood metabolic syndrome, subclinical atherosclerosis, and type 2 diabetes mellitus but is no better than body mass index alone: the Bogalusa Heart Study and the Cardiovascular Risk in Young Finns Study. Circulation. 2010;122(16):1604-1611. doi:10.1161/CIRCULATIONAHA.110.940809 20921439 PMC3388503

[42] Wang LY, Denniston M, Lee S, Galuska D, Lowry R. Long-term health and economic impact of preventing and reducing overweight and obesity in adolescence. J Adolesc Health. 2010;46(5):467-473. doi:10.1016/j.jadohealth.2009.11.204 20413083

[43] Jimenez MP, Aris IM, Rifas-Shiman S, . Early life exposure to greenness and executive function and behavior: an application of inverse probability weighting of marginal structural models. Environ Pollut. 2021;291:118208. doi:10.1016/j.envpol.2021.118208 34740291 PMC9208930

[44] Green MJ, Stritzel H, Smith C, Popham F, Crosnoe R. Timing of poverty in childhood and adolescent health: evidence from the US and UK. Soc Sci Med. 2018;197:136-143. doi:10.1016/j.socscimed.2017.12.004 29232621 PMC5777828

[45] Jimenez MP, Wellenius GA, Subramanian SV, . Longitudinal associations of neighborhood socioeconomic status with cardiovascular risk factors: a 46-year follow-up study. Soc Sci Med. 2019;241:112574. doi:10.1016/j.socscimed.2019.112574 31593787 PMC6913883

[46] Colombo J, Gustafson KM, Carlson SE. Critical and sensitive periods in development and nutrition. Ann Nutr Metab. 2019;75(suppl 1):34-42. doi:10.1159/000508053 32554960 PMC7393776

[47] Katzow M, Messito MJ, Mendelsohn AL, Scott MA, Gross RS. The protective effect of prenatal social support on infant adiposity in the first 18 months of life. J Pediatr. 2019;209:77-84. doi:10.1016/j.jpeds.2019.02.017 30879731 PMC6535345

[48] Hills AP, Andersen LB, Byrne NM. Physical activity and obesity in children. Br J Sports Med. 2011;45(11):866-870. doi:10.1136/bjsports-2011-090199 21836171

[49] Kim Y, Ritchie L, Landgraf A, Hasson RE, Colabianchi N. The role of the neighborhood social environment in physical activity among Hispanic children: moderation by cultural factors and mediation by neighborhood norms. Int J Environ Res Public Health. 2020;17(24):9527. doi:10.3390/ijerph17249527 33352648 PMC7766550

[50] Taverno SE, Rollins BY, Francis LA. Generation, language, body mass index, and activity patterns in Hispanic children. Am J Prev Med. 2010;38(2):145-153. doi:10.1016/j.amepre.2009.09.041 20117570 PMC2828268

[51] Bell CN, Thorpe RJ Jr, Laveist TA. Race/ethnicity and hypertension: the role of social support. Am J Hypertens. 2010;23(5):534-540. doi:10.1038/ajh.2010.28 20186126 PMC3102008

[52] Wu AJ, Aris IM, Hivert MF, . Association of changes in obesity prevalence with the COVID-19 pandemic in youth in Massachusetts. JAMA Pediatr. 2022;176(2):198-201. doi:10.1001/jamapediatrics.2021.5095 34901998 PMC8669600

[53] Boeke CE, Oken E, Kleinman KP, Rifas-Shiman SL, Taveras EM, Gillman MW. Correlations among adiposity measures in school-aged children. BMC Pediatr. 2013;13:99. doi:10.1186/1471-2431-13-99 23799991 PMC3693882

[54] Veitch J, van Stralen MM, Chinapaw MJM, . The neighborhood social environment and body mass index among youth: a mediation analysis. Int J Behav Nutr Phys Act. 2012;9:31. doi:10.1186/1479-5868-9-31 22429957 PMC3331800

[55] Jackson DB, Posick C, Vaughn MG. New evidence of the nexus between neighborhood violence, perceptions of danger, and child health. Health Aff (Millwood). 2019;38(5):746-754. doi:10.1377/hlthaff.2018.05127 31059369

[56] Chetty R, Hendren N, Katz LF. The effects of exposure to better neighborhoods on children: new evidence from the moving to opportunity experiment. Am Econ Rev. 2016;106(4):855-902. doi:10.1257/aer.20150572 29546974

[57] Frieden TR, Dietz W, Collins J. Reducing childhood obesity through policy change: acting now to prevent obesity. Health Aff (Millwood). 2010;29(3):357-363. doi:10.1377/hlthaff.2010.0039 20194973

