# Supplementary material for: Associations of Neighborhood Opportunity and Social Vulnerability With Trajectories of Childhood Body Mass Index and Obesity Among US Children
Source: JAMA Netw Open. 2022 Dec 22;5(12):e2247957. doi: 10.1001/jamanetworkopen.2022.47957 (PMC9857328; doi:10.1001/jamanetworkopen.2022.47957)
Supplement: Supplement 1. — eTable 1. Child Opportunity Index Indicators, Definitions, and Data Sources eTable 2. Social Vulnerability Index Indicators, Definitions, and Data Sources eTable 3. Child Opportunity Index and Rurality of Residence at Each Life Stage eTable 4. Social Vulnerability Index at Each Life Stage eTable 5. Participant Characteristics According to Very Low and Very High Child Opportunity Index (ChOI) Categories at Each Life Stage eTable 6. Participant Characteristics According to Very Low and Very High Social Vulnerability Index (SVI) Categories at Each Life Stage eTable 7. Overall F Statistic Values for Child Opportunity Index (ChOI) and Social Vulnerability Index (SVI) Models at Each Life Stage eFigure 1. Recruitment Site Locations for Cohorts Included in the Analytic Sample eFigure 2. Association of Child Opportunity Index Categories at Different Life Stages With Mean Difference in Body Mass Index and Risk of Obesity at Ages 0.5, 2, 5, 10, 15, and 20 Years eFigure 3. Association of Social Vulnerability Index Categories at Different Life Stages With Mean Difference in Body Mass Index and Risk of Obesity at Ages 0.5, 2, 5, 10, 15, and 20 Years eFigure 4. Association of Domain-Specific Child Opportunity Index Categories at Different Life Stages With Mean Difference in Body Mass Index and Risk of Obesity at Ages 10 and 15 Years eFigure 5. Association of Domain-Specific Social Vulnerability Index Categories at Different Life Stages With Mean Difference in Body Mass Index and Risk of Obesity at Ages 10 and 15 Years eFigure 6. Association of Child Opportunity Index Categories at Different Life Stages With Mean Difference in Body Mass Index and Risk of Obesity at Ages 0.5, 2, 5, 10, 15, and 20 Years Restricted to Residential Addresses Obtained During or After Year 2010 eFigure 7. Association of Child Opportunity Index Categories at Different Life Stages With Mean Difference in Body Mass Index and Risk of Obesity at Ages 10 and 15 Years by Child’s Race and Hispanic Ethnicity eFigu [file jamanetwopen-e2247957-s001.pdf]

Supplemental Online Content

Aris IM, Perng W, Dabelea D, et al; Program Collaborators for Environmental Influences on Child Health Outcomes. Associations of neighborhood opportunity and social vulnerability with trajectories of childhood body mass index and obesity among US children. *JAMA Netw Open*. 2022;5(12):e2247957. doi:10.1001/jamanetworkopen.2022.47957

eTable 1. Child Opportunity Index Indicators, Definitions, and Data Sources

eTable 2. Social Vulnerability Index Indicators, Definitions, and Data Sources

eTable 3. Child Opportunity Index and Rurality of Residence at Each Life Stage

eTable 4. Social Vulnerability Index at Each Life Stage

eTable 5. Participant Characteristics According to Very Low and Very High Child Opportunity Index (ChOI) Categories at Each Life Stage

eTable 6. Participant Characteristics According to Very Low and Very High Social Vulnerability Index (SVI) Categories at Each Life Stage

eTable 7. Overall *F* Statistic Values for Child Opportunity Index (COI) and Social Vulnerability Index (SVI) Models at Each Life Stage

eFigure 1. Recruitment Site Locations for Cohorts Included in the Analytic Sample

eFigure 2. Association of Child Opportunity Index Categories at Different Life Stages With Mean Difference in Body Mass Index and Risk of Obesity at Ages 0.5, 2, 5, 10, 15, and 20 Years

eFigure 3. Association of Social Vulnerability Index Categories at Different Life Stages With Mean Difference in Body Mass Index and Risk of Obesity at Ages 0.5, 2, 5, 10, 15, and 20 Years

eFigure 4. Association of Domain-Specific Child Opportunity Index Categories at Different Life Stages With Mean Difference in Body Mass Index and Risk of Obesity at Ages 10 and 15 Years

eFigure 5. Association of Domain-Specific Social Vulnerability Index Categories at Different Life Stages With Mean Difference in Body Mass Index and Risk of Obesity at Ages 10 and 15 Years

eFigure 6. Association of Child Opportunity Index Categories at Different Life Stages With Mean Difference in Body Mass Index and Risk of Obesity at Ages 0.5, 2, 5, 10, 15, and 20 Years Restricted to Residential Addresses Obtained During or After Year 2010

eFigure 7. Association of Child Opportunity Index Categories at Different Life Stages With Mean Difference in Body Mass Index and Risk of Obesity at Ages 10 and 15 Years by Child's Race and Hispanic Ethnicity

eFigure 8. Association of Social Vulnerability Index Categories at Different Life Stages With Mean Difference in Body Mass Index and Risk of Obesity at Ages 10 and 15 Years by Child's Race and Hispanic Ethnicity

eMethods. Procedure for Estimating Body Mass Index Trajectories

eReferences

This supplemental material has been provided by the authors to give readers additional information about their work.

© 2022 Aris IM et al. *JAMA Network Open*.

eTable 1. Child Opportunity Index Indicators, Definitions, and Data Sources[a]

Indicators	Definition (Data source and Year)
Education domain	
1. Early Childhood Education (ECE) centers	Number of ECE centers within a 5-mile radius (own data collection from state and federal sources; 2010)
2. High-quality ECE centers	Number of National Association for the Education of Young Children accredited centers within a 5-mile radius (own data collection from state and federal sources; 2010)
3. ECE enrollment	Percent 3- and 4-year-olds enrolled in nursery school, preschool or kindergarten (ACS; 2008–2012)
4. Third grade reading proficiency	Percent third graders scoring proficient on standardized reading tests, converted to NAEP scale score points (EDFacts, GS and SEDA; 2010)
5. Third grade math proficiency	Percent third graders scoring proficient on standardized math tests, converted to NAEP scale score points (EDFacts, GS and SEDA; 2010)
6. High school graduation rate	Percent ninth graders graduating from high school on time (EDFacts and GS; 2010)
7. Advanced Placement (AP) course enrollment	Ratio of students enrolled in at least one AP course to the number of 11th and 12th graders (CRDC; 2011)
8. College enrollment in nearby institutions	Percent 18-24 year-olds enrolled in college within 25-mile radius (ACS; 2008–2012)
9. School poverty	Percent students in elementary schools eligible for free or reduced-price lunches, reversed (NCES CCD; 2010)
10. Teacher experience[b]	Percent teachers in their first and second year (CRDC; 2011)
11. Adult educational attainment	Percent adults ages 25 and over with a college degree or higher (ACS; 2008–2012)
Health and Environment domain	
1. Access to healthy food[b]	Percent households without a car located further than a half-mile from the nearest super-market, reversed (USDA; 2010)
2. Access to green space[b]	Percent impenetrable surface areas such as rooftops, roads or parking lots (CDC; 2011)
3. Walkability	EPA Walkability Index (EPA; 2010–2012)
4. Housing vacancy rate[b]	Percent housing units that are vacant (ACS; 2008–2012)
5. Hazardous waste dump sites[b]	Average number of Superfund sites within a 2-mile radius (EPA; 2010)
6. Industrial pollutants in air, water or soil[b]	Index of toxic chemicals released by industrial facilities (EPA; 2010)
7. Airborne microparticles[b]	Mean estimated microparticle (PM2.5) concentration (CDC; 2010)
8. Ozone concentration[b]	Mean estimated 8-hour average ozone concentration (EPA; 2011)
9. Extreme heat exposure[b]	Summer days with maximum temperature above 90 degrees Fahrenheit (CDC; 2009–2011)
10. Health insurance coverage	Percent individuals ages 0-64 with health insurance coverage (ACS; 2008–2012)
Social and Economic domain	
1. Employment rate	Percent adults ages 25-54 who are employed (ACS; 2008–2012)
2. Commute duration[b]	Percent workers commuting more than one hour one way (ACS; 2008–2012)
3. Poverty rate[b]	Percent individuals living in households with incomes below 100% of the federal poverty threshold (ACS; 2008–2012)

© 2022 Aris IM et al. *JAMA Network Open*.

4. Public assistance rate[b]	Percent households receiving cash public assistance or Food Stamps/Supplemental Nutrition Assistance Program (ACS; 2008–2012)
5. Home ownership rate	Percent owner-occupied housing units (ACS; 2008–2012)
6. High-skill employment	Percent individuals ages 16 and over employed in management, business, financial, computer, engineering, science, education, legal, community service, health care practitioner, health technology, arts and media occupations (ACS; 2008–2012)
7. Median household income	Median income of all households (ACS; 2008–2012)
8. Single-headed households[b]	Percent family households that are single-parent headed (ACS; 2008–2012)

[a] Adapted from the Child Opportunity Index: Technical Documentation (see reference #20).
[b] Scores are reversed, such that a higher score for the indicator reflects higher opportunity levels.
ACS: American Community Survey; CDC: Center for Disease Control and Prevention; CRDC: U.S. Department of Education Office for Civil Rights Data Collection; EDFacts: U.S. Department of Education Data Files; EPA: Environmental Protection Agency; GS: Great Schools data; NAEP: National Assessment of Educational Progress; NCES CCD: National Center for Education Statistics Common Core of Data; SEDA: Stanford Education Data Archive; USDA: U.S. Department of Agriculture.

© 2022 Aris IM et al. *JAMA Network Open*.

eTable 2. Social Vulnerability Index Indicators, Definitions, and Data Sources

**Indicators**	**Definition (Data source)**
Socioeconomic	
1. Poverty	Persons below poverty estimate (ACS; 2000–2018)
2. Unemployment	Civilian (age 16+) unemployed estimate (ACS; 2000–2018)
3. Income	Per capita income estimate (ACS; 2000–2018)
4. High School Diploma	Persons (age 25+) with no high school diploma estimate (ACS; 2000–2018)
Household Composition and Disability	
1. Aged 65 or Older	Persons aged 65 and older estimate (ACS; 2000–2018)
2. Aged 17 or Younger	Persons aged 17 and younger estimate (ACS; 2000–2018)
3. Civilian with a Disability	Civilian noninstitutionalized population with a disability estimate (ACS; 2000–2018)
4. Single-Parent Households	Single parent household with children under 18 estimate (ACS; 2000–2018)
Minority Status and Language	
1. Minority	Minority (all persons except white, non-Hispanic) estimate (ACS; 2000–2018)
2. Speaks English "Less than Well"	Persons (age 5+) who speak English "less than well" estimate (ACS; 2000–2018)
Housing and Transportation	
1. Multi-Unit Structures	Housing in structures with 10 or more units estimate (ACS; 2000–2018)
2. Mobile Homes	Mobile homes estimate (ACS; 2000–2018)
3. Crowding	At household level (occupied housing units), more people than rooms estimate (ACS; 2000–2018)
4. No Vehicle	Households with no vehicle available estimate (ACS; 2000–2018)
5. Group Quarters	Persons in institutionalized and noninstitutionalized group quarters estimate (ACS; 2000–2018)

ACS: American Community Survey.

© 2022 Aris IM et al. *JAMA Network Open*.

eTable 3. Child Opportunity Index and Rurality of Residence at Each Life Stage

	**Birth (n = 18,044)**	**Infancy (n = 17,300)**	**Early childhood (n = 15,452)**	**Mid-childhood (n = 11,190)**
Child Opportunity Index	**Mean (SD) or %**			
**Overall**				
. Mean percentile rank	54.0 (30.5)	54.8 (30.6)	56.0 (30.6)	57.4 (30.1)
. Very Low opportunity	20.8	20.0	18.9	16.9
. Low opportunity	14.7	14.7	14.3	14.3
. Moderate opportunity	16.0	15.7	15.8	16.2
. High opportunity	21.8	21.8	21.6	21.9
. Very High opportunity	26.7	27.8	29.4	30.6
**Education**				
. Mean percentile rank	53.6 (28.3)	54.0 (28.5)	54.0 (28.8)	54.1 (29.1)
. Very Low opportunity	18.5	18.3	18.2	18.0
. Low opportunity	15.3	15.0	15.4	16.0
. Moderate opportunity	19.7	19.6	19.6	19.4
. High opportunity	25.7	25.3	24.0	23.1
. Very High opportunity	20.9	21.8	22.7	23.6
**Health and Environment**				
. Mean percentile rank	57.5 (30.0)	58.6 (30.2)	60.1 (30.4)	61.7 (30.1)
. Very Low opportunity	16.0	15.5	15.0	14.0
. Low opportunity	15.7	14.9	14.0	12.4
. Moderate opportunity	17.0	16.6	16.0	15.7
. High opportunity	20.8	20.2	19.4	20.7
. Very High opportunity	30.5	32.8	35.6	37.2
**Social and Economic**				
. Mean percentile rank	53.6 (31.2)	54.4 (31.2)	55.6 (31.0)	57.2 (30.4)
. Very Low opportunity	21.6	20.8	19.3	17.1
. Low opportunity	15.6	15.5	15.2	14.7
. Moderate opportunity	14.9	14.7	15.3	16.1
. High opportunity	20.4	20.8	21.2	21.8
. Very High opportunity	27.4	28.2	29.1	30.2
Rural/urban categories				
. Metropolitan	85.7	85.9	85.7	84.4
. Micropolitan	7.0	7.0	7.7	8.8
. Small town/rural areas	7.3	7.1	6.6	6.8
Child body mass index				
. Mean body mass index	13.1 (1.6)	17.3 (1.7)	16.4 (1.8)	17.2 (3.3)
. Obesity prevalence	5.1	14.5	11.5	15.1

© 2022 Aris IM et al. *JAMA Network Open*.

eTable 4. Social Vulnerability Index at Each Life Stage

	Birth (n =18,044)	Infancy (n =17,300)	Early childhood (n =15,452)	Mid-childhood (n =11,190)
	Mean (SD) or %			
**Overall**				
. Mean percentile rank	0.47 (0.31)	0.47 (0.30)	0.46 (0.30)	0.44 (0.30)
. Very High vulnerability	21.1	20.5	19.3	16.9
. High vulnerability	17.0	16.6	16.2	16.5
. Moderate vulnerability	16.9	16.8	17.0	16.4
. Low vulnerability	19.8	20.0	20.6	21.7
. Very Low vulnerability	25.3	26.0	26.8	28.4
**Socioeconomic**				
. Mean percentile rank	0.44 (0.30)	0.44 (0.30)	0.43 (0.30)	0.42 (0.29)
. Very High vulnerability	18.2	17.7	16.8	14.6
. High vulnerability	15.3	15.1	15.5	16.4
. Moderate vulnerability	15.8	16.2	16.0	16.4
. Low vulnerability	21.0	20.8	21.2	21.6
. Very Low vulnerability	29.6	30.3	30.6	31.0
**Household composition**				
. Mean percentile rank	0.44 (0.29)	0.44 (0.29)	0.45 (0.28)	0.47 (0.28)
. Very High vulnerability	15.9	15.8	15.2	16.1
. High vulnerability	15.7	16.4	17.3	18.5
. Moderate vulnerability	19.3	19.2	19.6	20.1
. Low vulnerability	23.3	23.4	23.9	24.6
. Very Low vulnerability	25.9	25.3	23.9	20.8
**Minority status/language**				
. Mean percentile rank	0.54 (0.31)	0.54 (0.31)	0.52 (0.30)	0.48 (0.29)
. Very High vulnerability	27.8	26.6	23.7	18.8
. High vulnerability	20.1	20.0	20.1	20.1
. Moderate vulnerability	16.7	17.0	17.5	18.5
. Low vulnerability	15.7	16.4	17.6	19.9
. Very Low vulnerability	19.7	19.9	21.1	22.8
**Housing type/Transportation**				
. Mean percentile rank	0.53 (0.30)	0.52 (0.30)	0.50 (0.29)	0.47 (0.29)
. Very High vulnerability	24.3	22.8	20.8	17.3
. High vulnerability	22.2	21.5	19.9	19.0
. Moderate vulnerability	18.4	18.9	20.2	21.0
. Low vulnerability	16.1	16.8	17.7	19.2
. Very Low vulnerability	19.0	20.0	21.4	23.5

© 2022 Aris IM et al. *JAMA Network Open*.

eTable 5. Participant Characteristics According to Very Low and Very High Child Opportunity Index (ChOI) Categories at Each Life Stage

	Birth		Infancy		Early childhood		Mid-childhood	
**Overall ChOI categories**	**Very low N = 3,749**	**Very high N = 4,814**	**Very low N = 3,454**	**Very high N = 4,814**	**Very low N = 2,921**	**Very high N = 4,540**	**Very low N = 1,896**	**Very high N = 3,249**
**Domain specific ChOI percentile rank**	**Mean (SD) or %**		**Mean (SD) or %**		**Mean (SD) or %**		**Mean (SD) or %**	
. Education	17.4 (14.0)	82.2 (11.9)	17.3 (14.1)	82.4 (12.0)	16.9 (14.0)	82.7 (12.4)	15.0 (13.8)	83.0 (12.7)
. Health and Environment	21.1 (21.3)	83.9 (15.4)	21.2 (21.4)	84.7 (15.3)	21.8 (22.1)	85.5 (14.8)	22.5 (23.8)	85.9 (14.2)
. Social and Economic	9.8 (6.7)	89.1 (7.2)	9.7 (6.7)	89.1 (7.2)	9.6 (6.6)	89.2 (7.6)	9.6 (6.9)	89.1 (7.7)
**Rurality of residence**								
. Metropolitan	96.3	83.2	96.2	83.0	95.8	83.5	94.1	83.8
. Micropolitan	1.9	8.4	1.8	8.3	2.2	8.4	3.2	8.4
. Small town/rural areas	1.8	8.4	1.9	8.7	2.0	8.0	2.7	7.8
**Birth year**								
. Before 2000	0.3	3.5	0.3	3.6	0.2	4.0	0.8	6.3
. 2000 to 2010	34.6	23.3	36.3	24.0	41.1	27.8	55.4	32.9
. After 2010	65.1	73.2	63.5	72.4	58.7	68.2	43.8	60.8
**Child sex**								
. Male	51.3	50.6	50.9	50.7	50.3	50.5	51.4	51.1
. Female	48.7	49.4	49.1	49.3	49.7	49.5	48.6	48.9
**Child race**								
. White	32.8	75.8	32.3	75.6	28.8	74.8	20.3	75.0
. Black	44.3	3.1	45.1	3.1	49.0	3.4	57.9	4.2
. Asian	1.6	6.2	1.5	6.2	1.5	6.4	1.6	4.8
. Native Hawaiian or Pacific Islander, American Indian or Alaska Native	2.5	0.5	2.5	0.5	2.4	0.6	2.4	0.6
. Other race or More than one race	18.7	14.5	18.6	14.6	18.3	14.9	17.9	15.5
**Hispanic ethnicity**	36.0	11.7	35.9	11.2	33.4	10.7	21.2	10.0
**Education level during pregnancy**								
. Less than high school	18.9	3.1	19.4	3.2	20.2	3.2	18.1	3.6
. High school degree or equivalent	32.8	8.6	33.7	8.7	34.5	8.6	34.8	8.8
. Some college, no degree	26.8	18.4	27.0	18.3	26.4	18.2	27.9	18.4
. College degree and above	21.6	69.9	19.9	69.8	18.9	70.0	19.1	69.2
**Household income during pregnancy**								
. < $50,000/year	78.9	28.7	80.5	29.0	81.9	28.9	81.8	30.8
. ≥ $50,000/year	21.1	71.3	19.5	71.0	18.1	71.1	18.2	69.2
**Cigarette smoking during pregnancy**	13.6	7.6	14.2	8.0	14.5	8.0	18.1	8.8
**Nulliparous**	35.1	42.0	35.6	41.5	34.9	42.0	35.7	40.6
**Gestational diabetes**	10.7	7.4	10.5	7.2	9.4	7.0	6.0	6.7
**Gestational hypertension/preeclampsia**	8.1	8.8	8.2	8.8	8.9	8.7	8.5	8.5
**Cesarean delivery**	33.6	26.9	32.8	27.2	32.4	27.7	32.5	28.3
**Pre-pregnancy body mass index (kg/m^2)**	29.0 (7.6)	25.4 (6.1)	29.0 (7.6)	25.5 (6.0)	28.9 (7.6)	25.4 (6.0)	28.8 (7.9)	25.5 (6.1)
**Total gestational weight gain (kg)**	13.3 (9.3)	16.6 (11.6)	13.4 (9.3)	16.7 (11.7)	13.7 (9.2)	16.5 (11.3)	14.1 (9.6)	16.6 (11.2)
**Gestational age at delivery (weeks)**	37.7 (3.7)	38.1 (4.5)	37.8 (3.6)	38.2 (4.4)	37.7 (3.8)	38.2 (4.5)	37.3 (4.3)	38.0 (5.0)
**Birth weight (kg)**	3.1 (0.8)	3.3 (0.7)	3.1 (0.8)	3.3 (0.7)	3.0 (0.8)	3.3 (0.7)	2.9 (0.9)	3.3 (0.7)

© 2022 Aris IM et al. *JAMA Network Open*.

eTable 6. Participant Characteristics According to Very Low and Very High Social Vulnerability Index (SVI) Categories at Each Life Stage

	Birth		Infancy		Early childhood		Mid-childhood	
**Overall SVI categories**	**Very high N = 3,809**	**Very low N = 4,559**	**Very high N = 3,554**	**Very low N = 4,504**	**Very high N = 2,981**	**Very low N = 4,144**	**Very high N = 1,896**	**Very low N = 3,179**
**Domain specific SVI percentile rank**	**Mean (SD) or %**		**Mean (SD) or %**		**Mean (SD) or %**		**Mean (SD) or %**	
. Socioeconomic	0.9 (0.1)	0.1 (0.1)	0.9 (0.1)	0.1 (0.1)	0.9 (0.1)	0.1 (0.1)	0.8 (0.1)	0.1 (0.1)
. Household composition	0.7 (0.2)	0.3 (0.2)	0.7 (0.2)	0.3 (0.2)	0.7 (0.2)	0.3 (0.2)	0.8 (0.2)	0.3 (0.2)
. Minority status/language	0.9 (0.2)	0.3 (0.2)	0.8 (0.2)	0.3 (0.2)	0.8 (0.2)	0.3 (0.2)	0.8 (0.2)	0.3 (0.2)
. Housing type/Transportation	0.8 (0.2)	0.2 (0.2)	0.8 (0.2)	0.2 (0.2)	0.8 (0.2)	0.2 (0.2)	0.8 (0.2)	0.2 (0.2)
**Rurality of residence**								
. Metropolitan	96.2	73.5	96.0	75.5	94.6	78.8	92.5	77.9
. Micropolitan	2.4	14.2	2.4	13.9	2.1	13.4	2.7	14.0
. Small town/rural areas	1.4	12.3	1.6	10.6	3.3	7.8	4.8	8.1
**Birth year**								
. Before 2000	0.5	3.8	0.6	3.7	0.5	4.0	0.6	5.8
. 2000 to 2010	29.8	29.7	29.9	30.9	35.3	33.2	48.9	35.2
. After 2010	69.7	66.5	69.5	65.4	64.2	49.3	50.4	59.0
**Child sex**								
. Male	52.7	50.9	51.8	51.2	50.9	50.7	51.6	50.1
. Female	47.3	49.1	48.2	48.8	49.1	49.3	48.4	49.9
**Child race**								
. White	38.2	81.3	38.3	79.5	35.7	79.0	26.4	79.4
. Black	36.2	4.6	36.5	5.0	39.2	4.9	48.3	4.4
. Asian	3.0	2.1	2.8	2.7	2.8	3.0	3.1	2.5
. Native Hawaiian or Pacific Islander, American Indian or Alaska Native	2.8	0.5	2.6	0.5	2.4	0.5	2.5	0.3
. Other race or More than one race	19.8	11.5	19.7	12.3	19.9	12.7	19.7	13.4
**Hispanic ethnicity**	43.0	8.1	43.0	7.9	40.0	8.7	27.6	7.8
**Education level during pregnancy**								
. Less than high school	17.3	3.5	17.9	3.5	19.1	3.5	17.2	3.9
. High school degree or equivalent	29.7	10.9	29.7	10.8	30.7	10.4	29.9	10.1
. Some college, no degree	28.8	20.0	29.3	19.9	28.4	19.8	30.7	19.1
. College degree and above	24.2	65.6	23.1	65.8	21.8	66.3	22.2	66.9
**Household income during pregnancy**								
. < $50,000/year	73.4	34.6	74.6	34.4	77.6	33.2	77.5	33.8
. ≥ $50,000/year	26.6	65.4	25.4	65.6	22.4	66.8	22.5	66.2
**Cigarette smoking during pregnancy**	11.6	10.0	12.1	10.0	13.5	9.4	16.7	9.0
**Nulliparous**	36.6	40.1	37.3	40.5	36.0	40.1	36.9	40.6
**Gestational diabetes**	10.6	7.0	10.9	7.0	9.8	6.5	5.7	6.7
**Gestational hypertension/preeclampsia**	7.9	9.2	8.2	9.3	8.9	8.8	8.5	8.8
**Cesarean delivery**	32.6	28.2	32.0	28.1	32.3	29.0	33.3	30.0
**Pre-pregnancy body mass index (kg/m^2)**	28.7 (7.3)	25.6 (6.1)	28.8 (7.3)	25.4 (5.9)	28.8 (7.6)	25.5 (6.0)	28.7 (7.8)	25.6 (6.2)
**Total gestational weight gain (kg)**	13.3 (9.1)	18.3 (12.6)	13.4 (9.3)	17.9 (12.4)	13.6 (9.5)	17.3 (11.7)	14.0 (9.6)	17.4 (11.9)
**Gestational age at delivery (weeks)**	37.7 (3.7)	38.3 (4.1)	37.8 (3.7)	38.3 (3.9)	37.7 (3.8)	38.2 (4.2)	37.4 (4.3)	38.0 (5.0)
**Birth weight (kg)**	3.1 (0.8)	3.3 (0.7)	3.1 (0.8)	3.3 (0.7)	3.0 (0.8)	3.3 (0.7)	3.0 (0.8)	3.3 (0.8)

© 2022 Aris IM et al. *JAMA Network Open*.

eTable 7. Overall *F* Statistic Values for Child Opportunity Index (ChOI) and Social Vulnerability Index (SVI) Models at Each Life Stage

		Outcome: Body mass index			Outcome: Obesity		
		**ChOI**	**SVI**	**% difference**	**ChOI**	**SVI**	**% difference**
Birth		17452.39	17416.19	0.21	313.79	310.13	1.17
Infancy		3666.48	3649.65	0.46	260.05	258.53	0.58
Early childhood		3130.48	3119.58	0.35	356.69	361.72	1.41
Mid-childhood		2182.53	2163.92	0.85	174.90	171.64	1.86

© 2022 Aris IM et al. *JAMA Network Open*.

eFigure 1. Recruitment Site Locations for Cohorts Included in the Analytic Sample

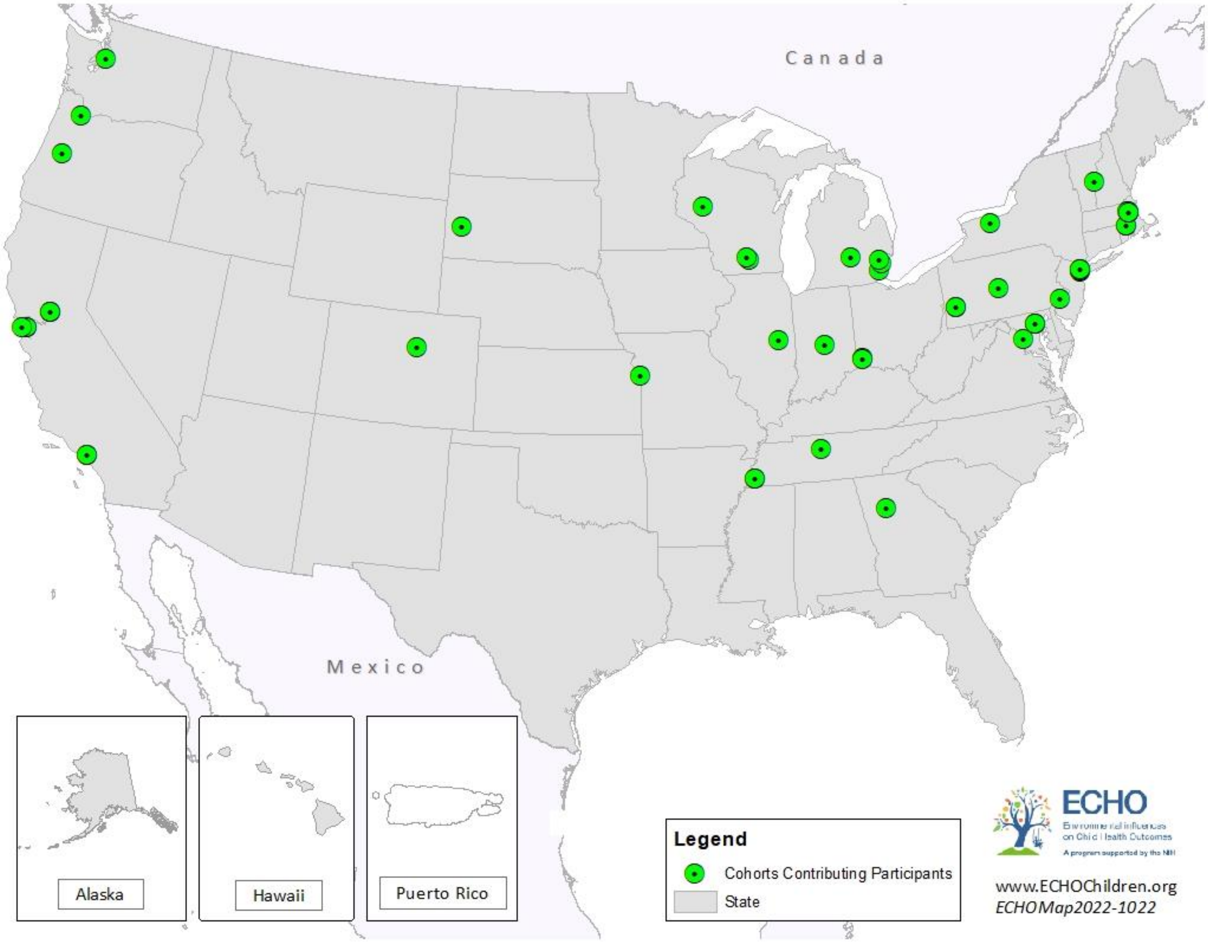

© 2022 Aris IM et al. *JAMA Network Open*.

eFigure 2. Association of Child Opportunity Index Categories at Different Life Stages With Mean Difference in Body Mass Index and Risk of Obesity at Ages 0.5, 2, 5, 10, 15, and 20 Years

Mean difference in body mass index (A-D) and risk of obesity (E-H). All effect estimates and 95% CIs are relative to the very low Child Opportunity Index (ChOI) category and adjusted for sociodemographic and prenatal characteristics and ChOI at previous life stages.

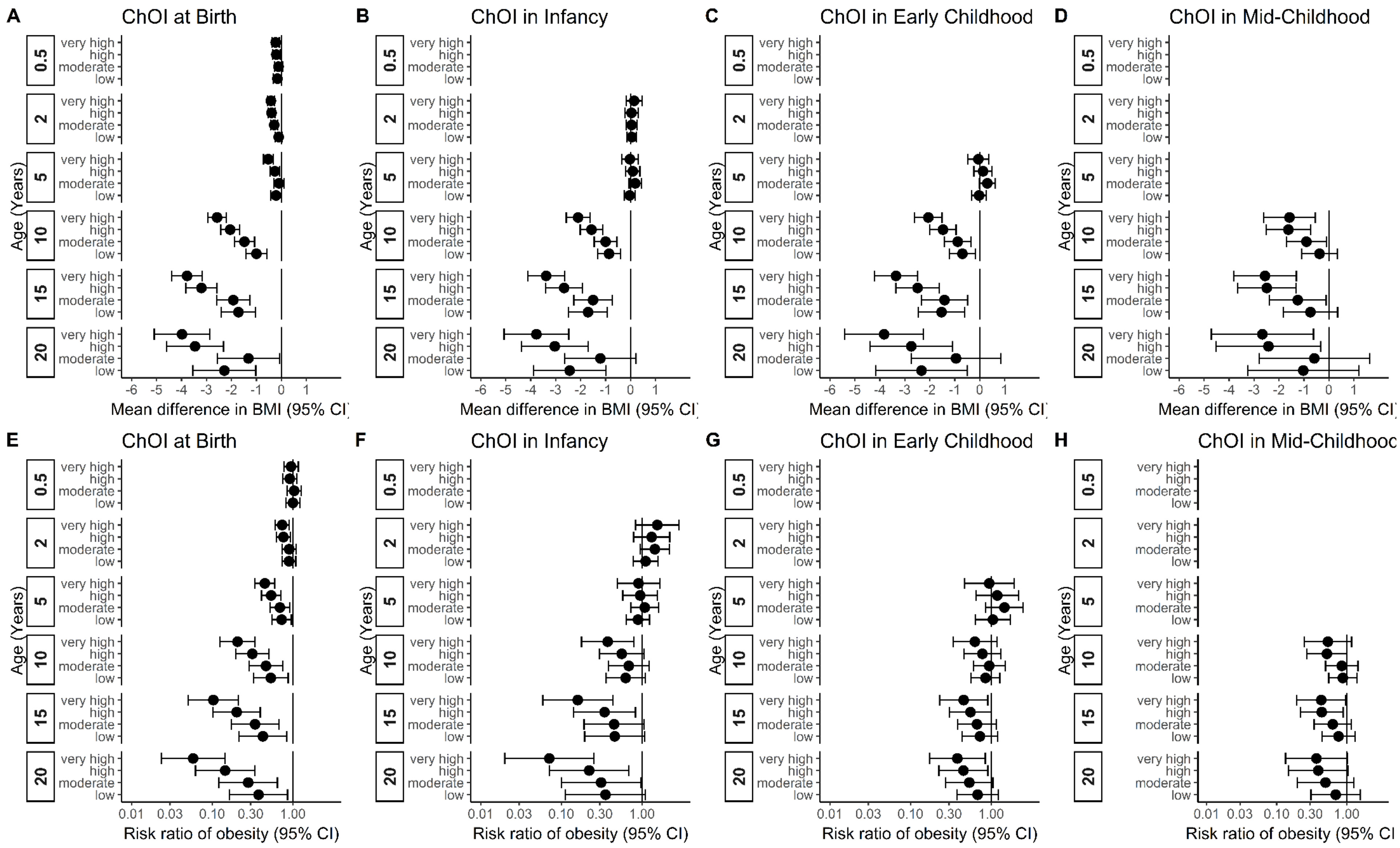

© 2022 Aris IM et al. *JAMA Network Open*.

eFigure 3. Association of Social Vulnerability Index Categories at Different Life Stages With Mean Difference in Body Mass Index and Risk of Obesity at Ages 0.5, 2, 5, 10, 15, and 20 Years

Mean difference in body mass index (A-D) and risk of obesity (E-H). All effect estimates and 95% CIs are relative to the very high Social Vulnerability Index (SVI) category and adjusted for sociodemographic and prenatal characteristics and SVI at previous life stages.

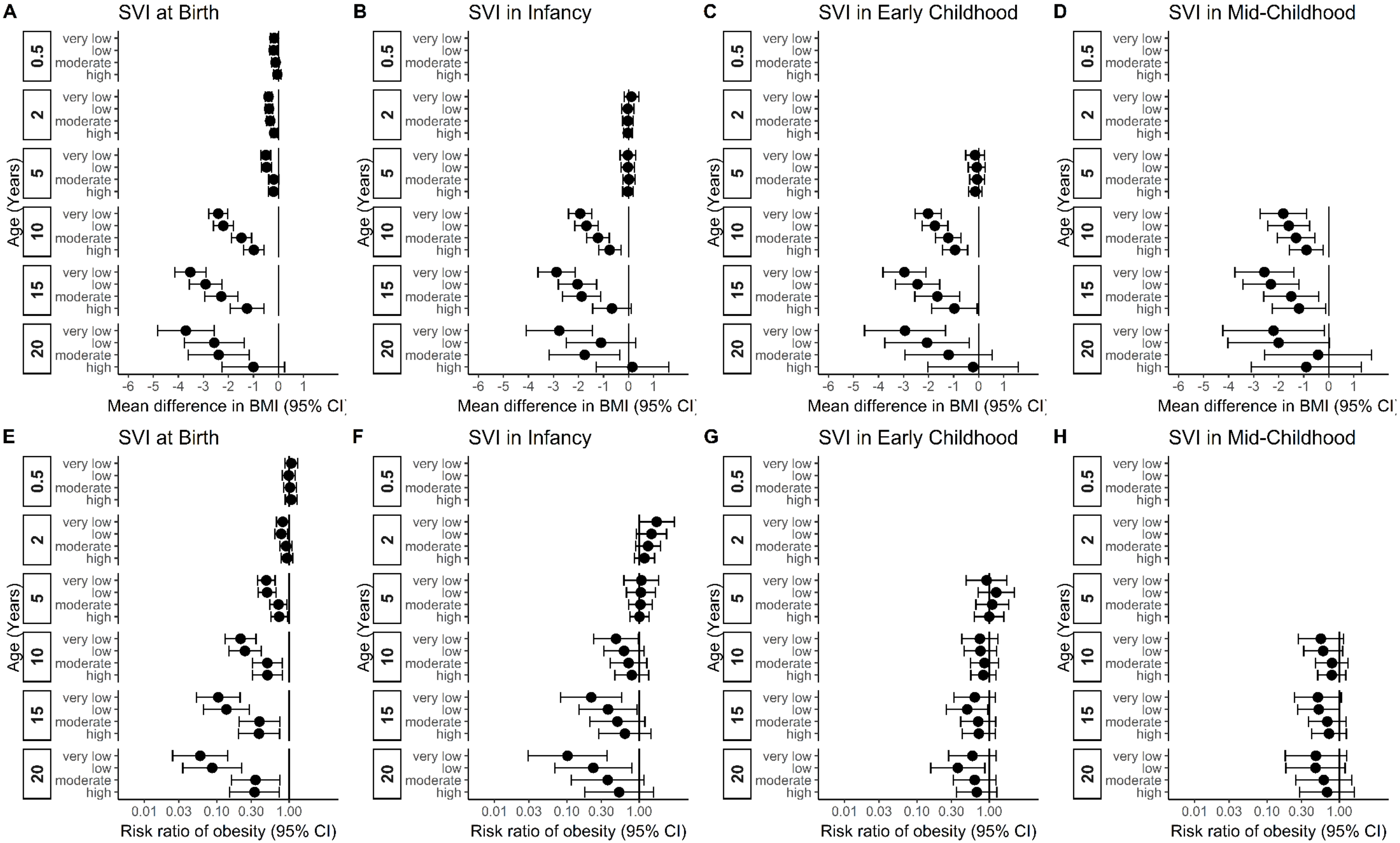

© 2022 Aris IM et al. *JAMA Network Open*.

eFigure 4. Association of Domain-Specific Child Opportunity Index Categories at Different Life Stages With Mean Difference in Body Mass Index and Risk of Obesity at Ages 10 and 15 Years

Mean difference in body mass index (A-D) and risk of obesity (E-H). All effect estimates and 95% CIs are relative to the very low Child Opportunity Index (ChOI) category and adjusted for sociodemographic and prenatal characteristics

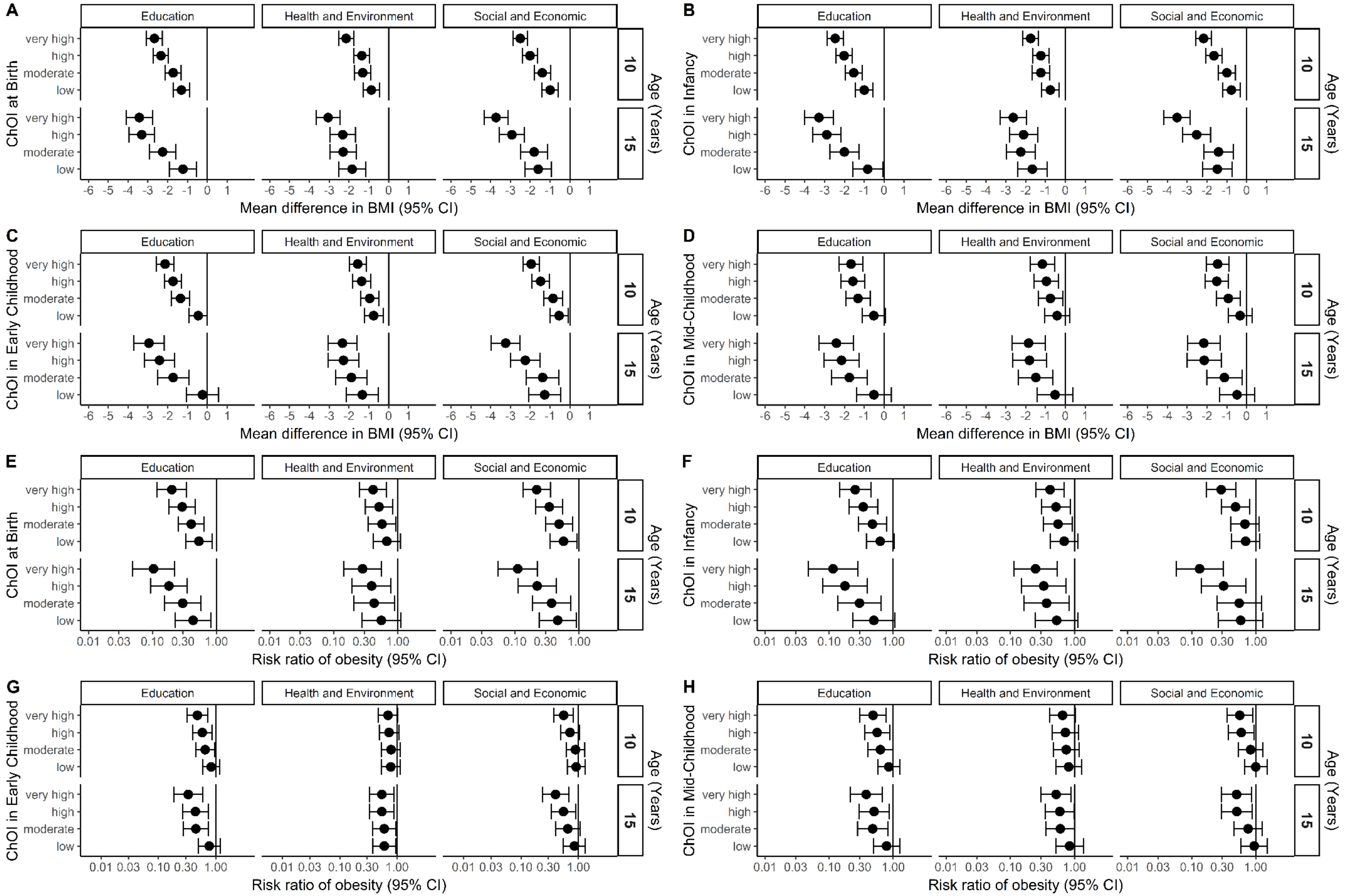

© 2022 Aris IM et al. *JAMA Network Open*.

eFigure 5. Association of Domain-Specific Social Vulnerability Index Categories at Different Life Stages With Mean Difference in Body Mass Index and Risk of Obesity at Ages 10 and 15 Years

Mean difference in body mass index (A-D) and risk of obesity (E-H). All effect estimates and 95% CIs are relative to the very high Social Vulnerability Index (SVI) category and adjusted for sociodemographic and prenatal characteristics.

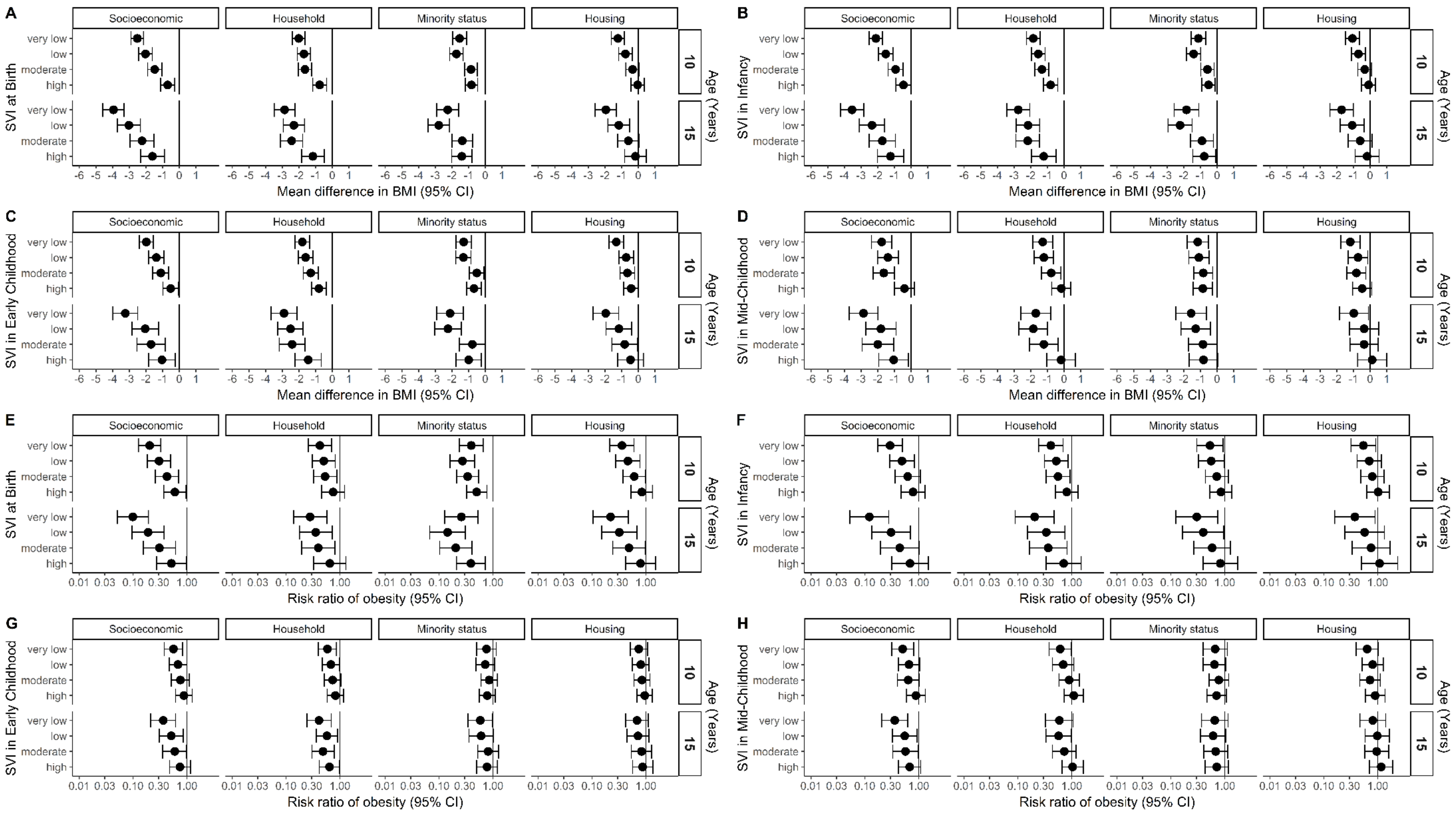

© 2022 Aris IM et al. *JAMA Network Open*.

eFigure 6. Association of Child Opportunity Index Categories at Different Life Stages With Mean Difference in Body Mass Index and Risk of Obesity at Ages 0.5, 2, 5, 10, 15, and 20 Years Restricted to Residential Addresses Obtained During or After Year 2010

Mean difference in body mass index (A-D) and risk of obesity (E-H). All effect estimates and 95% CIs are relative to the very low Child Opportunity Index (ChOI) category and adjusted for sociodemographic and prenatal characteristics.

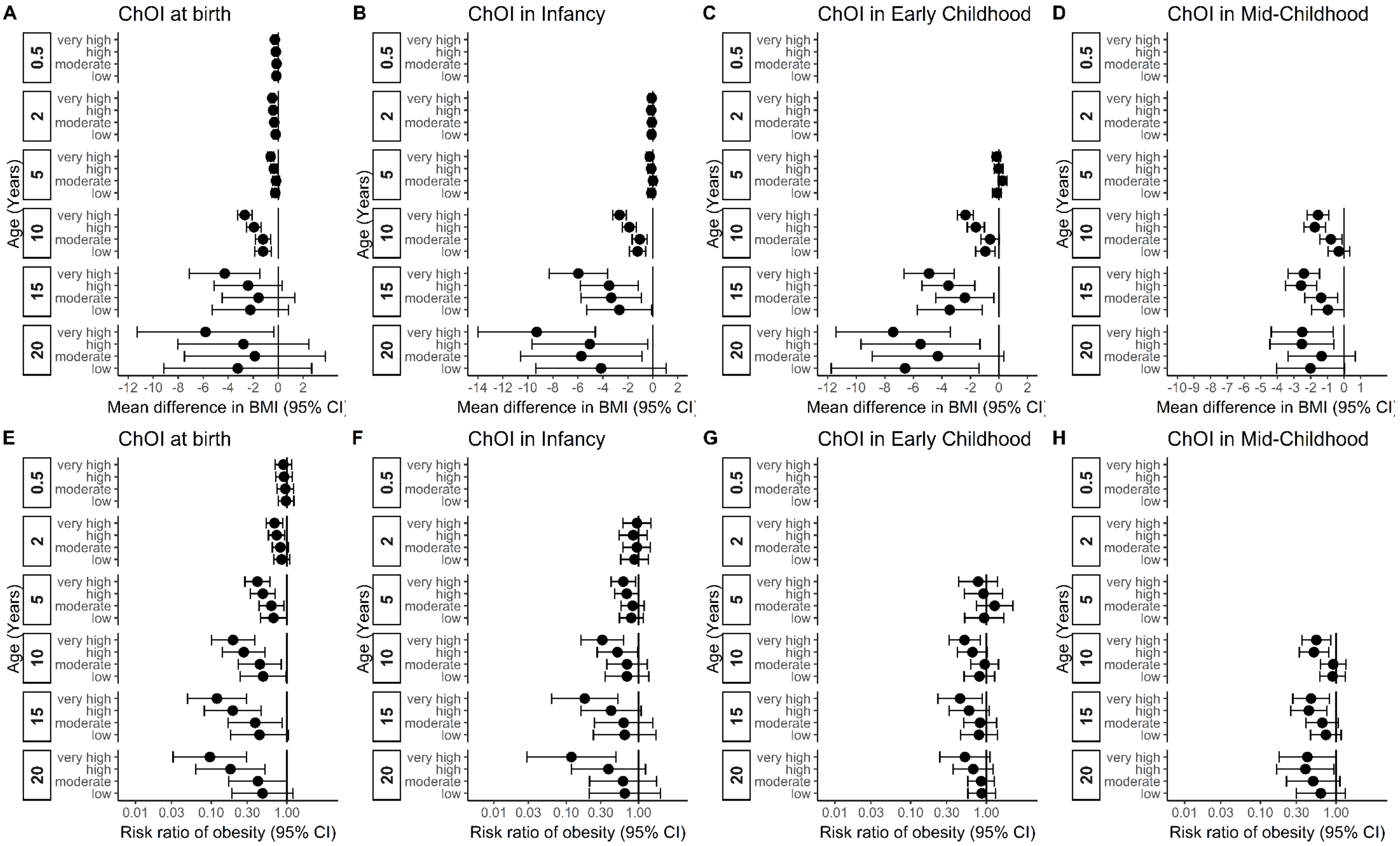

© 2022 Aris IM et al. *JAMA Network Open*.

eFigure 7. Association of Child Opportunity Index Categories at Different Life Stages With Mean Difference in Body Mass Index and Risk of Obesity at Ages 10 and 15 Years by Child's Race and Hispanic Ethnicity

All effect estimates and 95% CIs are relative to the very low Child Opportunity Index (ChOI) category and adjusted for sociodemographic and prenatal characteristics.

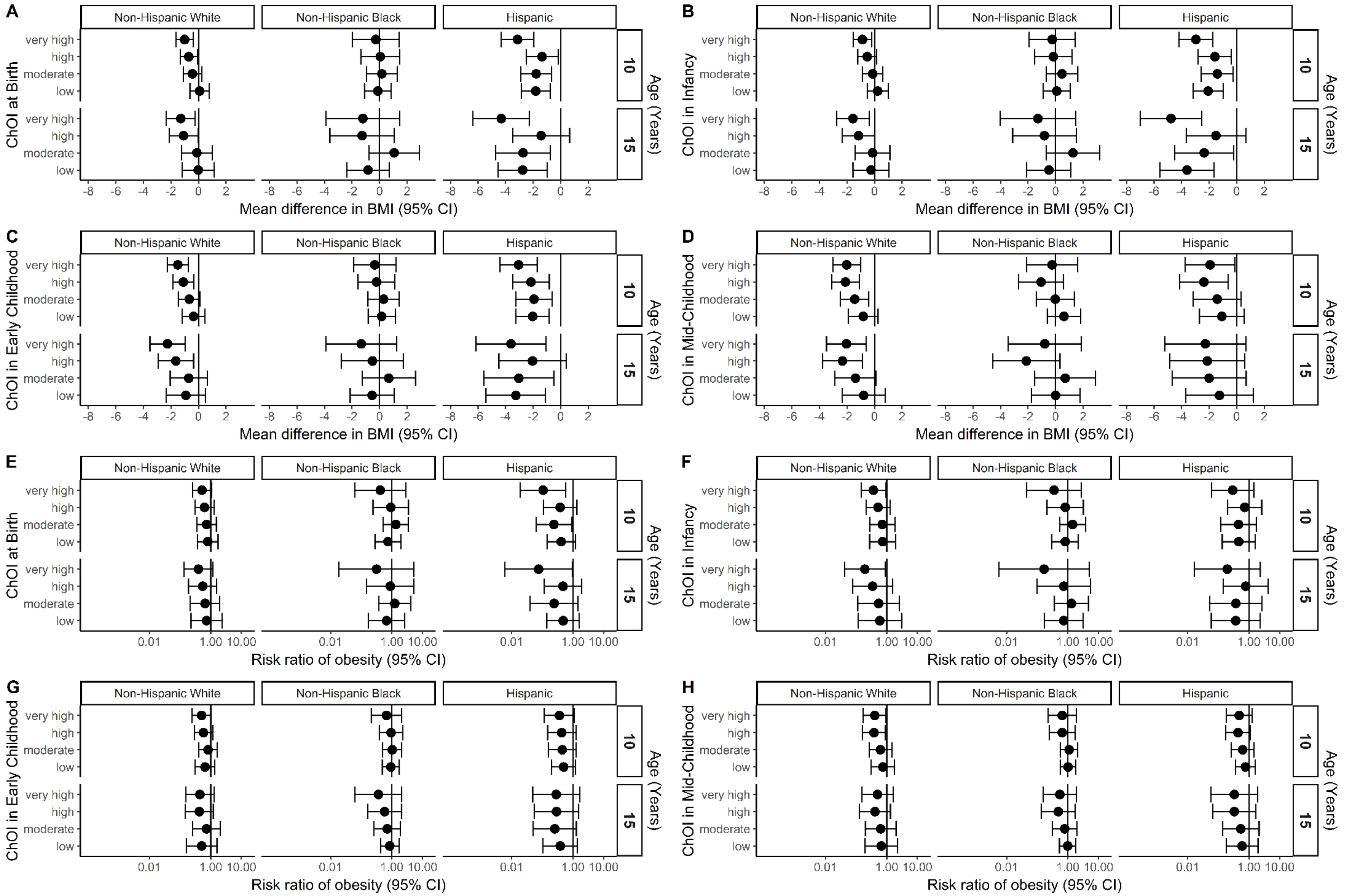

© 2022 Aris IM et al. *JAMA Network Open*.

eFigure 8. Association of Social Vulnerability Index Categories at Different Life Stages With Mean Difference in Body Mass Index and Risk of Obesity at Ages 10 and 15 Years by Child's Race and Hispanic Ethnicity

Mean difference in body mass index (A-D) and risk of obesity (E-H). All effect estimates and 95% CIs are relative to the very high Social Vulnerability Index (SVI) category and adjusted for sociodemographic and prenatal characteristics.

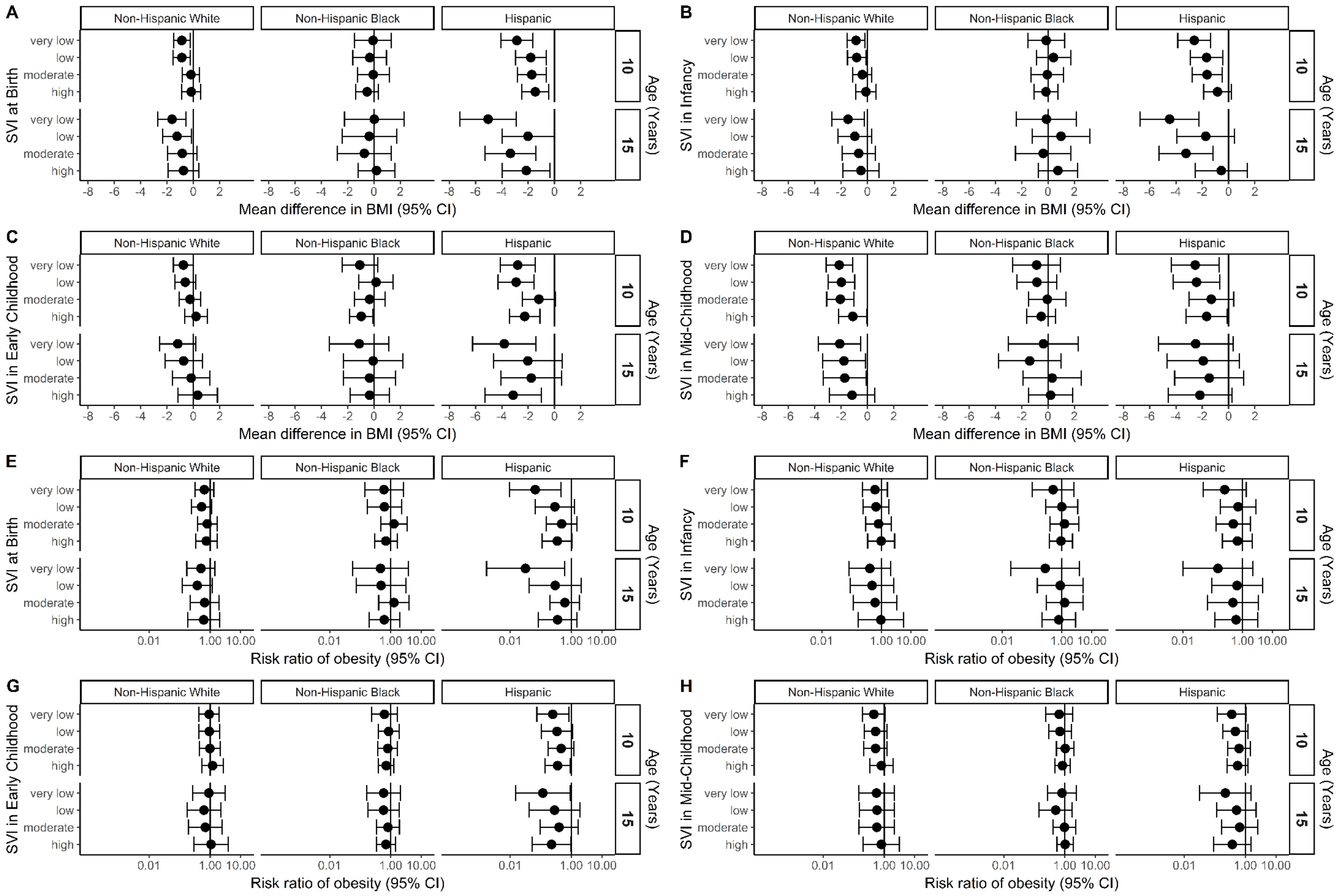

© 2022 Aris IM et al. *JAMA Network Open*.

eMethods. Procedure for Estimating Body Mass Index (BMI) Trajectories

We estimated trajectories of BMI from birth to adolescence for each neighborhood index category using linear mixed-effects models. Briefly, these models were fitted with maximum-likelihood estimation, a method of estimating the parameters of an assumed probability distribution given the observed data.[1] Specifically, this method allows us to obtain parameter estimates even in the presence of missing data. It does not impute any data but rather, uses each individual's available data to compute the maximum likelihood. Thus, these models enable estimation of BMI trajectories in children even if they only had one measure of BMI from birth to adolescence, with the caveat that children with a greater number of missing BMI measures would have BMI trajectories that are closer to the population average. Such models also allow for changes in scale and variance of BMI over time.[2] The fixed effects component of the model (without exposure or covariates) was as follows:

$$BMI_i = \beta_{0i} + \beta_{1i}\,(age) + \sum_{j=2}^{m} \beta_{ij}\{(age\text{-}k_j)^3{}_{+} - \lambda_j(age\text{-}k_{min})^3{}_{+} - (1\text{-}\ \lambda_j)(age\text{-}k_{max})^3{}_{+}\} + e_{ij}$$

where for child *i*, k_{min} and k_{max} = boundary knots; k_j = interior knot point *j* between boundary knots; λ_j = distance between knot points k_m and k_j; *m* = number of interior knots between boundary knots; *j* = 2, …, m; *e* = error; and $(age\text{-}k_j)^3{}_{+}$ = age−k if age ≥ k_j. The random effects component of the model contained random effects for the intercept and linear age slopes. We used an unstructured covariance matrix so that each random effect would be correlated with all other random effects. We considered two approaches to select interior knot locations: at equally spaced percentiles between birth and adolescence,[3] and using knowledge of underlying biology of BMI growth patterns.[4] We determined the optimal number (4 knots) and location (0.05, 1, 4.5, and 10 years) of interior knots using the Bayesian information criterion.

We also included the following variables as fixed effects: neighborhood index categories, interactions of neighborhood index categories with each spline term for child age, sociodemographics, and prenatal characteristics. From these models, we predicted the population average BMI over time for each neighborhood index category and plotted the corresponding BMI trajectory, holding all covariates constant at their mean values.

© 2022 Aris IM et al. *JAMA Network Open*.

eReferences

1. Verbeke G. Linear Mixed Models for Longitudinal Data. In: Linear Mixed Models in Practice, Springer, New York. 1997;126:63-153.
2. Goldstein H. Efficient statistical modelling of longitudinal data. Ann Hum Biol. 1986;13(2):129-141.
3. Harrell Jr FE. Regression modeling strategies: with applications to linear models, logistic and ordinal regression, and survival analysis. Springer; 2015.
4. Wen X, Kleinman K, Gillman MW, Rifas-Shiman SL, Taveras EM. Childhood body mass index trajectories: modeling, characterizing, pairwise correlations and socio-demographic predictors of trajectory characteristics. BMC Med Res Methodol. 2012;12:38.

© 2022 Aris IM et al. *JAMA Network Open*.
