# Supplementary material for: Associations of Neighborhood Opportunity and Social Vulnerability With Trajectories of Childhood Body Mass Index and Obesity Among US Children
Source: JAMA Netw Open. 2022 Dec 22;5(12):e2247957. doi: 10.1001/jamanetworkopen.2022.47957 (PMC9857328; doi:10.1001/jamanetworkopen.2022.47957)
Supplement: Supplement 2. — Nonauthor Collaborators. The Program Collaborators for Environmental Influences on Child Health Outcomes [file jamanetwopen-e2247957-s002.pdf]

Supplemental Online Content: Nonauthor Collaborators
*First name, last name, and suffix (if applicable) are required and will appear in PubMed.

*Group Name(s): Program Collaborators for Environmental influences on Child Health Outcomes							
***First Name and Middle Initial(s)**	***Last Name**	***Suffix (eg, Jr, III)**	Academic Degrees	Institution	Location (city, state/province, country)	Role or Contribution, eg, chair, principal investigator	Group (if more than 1 Group listed in the byline) and/or Subgroup (eg, Steering Committee)
Samia	Baluch			Duke Clinical Research Institute	Durham, NC	Coordinating Center	
Timothy	Shields			Johns Hopkins University Bloomberg School of Public Health	Baltimore, MD	Coordinating Center	
Stephanie	Merhar			Cincinnati Children's Hospital Medical Center	Cincinnati, Ohio	Principal Investigator	
Clement	Ren			Indiana University, Riley Hospital for Children	Indianapolis, IN	Principal Investigator	
Gloria	Pryhuber			University of Rochester Medical Center	Rochester, NY	Principal Investigator	
Paul	Moore			Vanderbilt Children's Hospital	Nashville, TN	Principal Investigator	
Rajan	Wadhawan			Florida Hospital for Children	Orlando, FL	Principal Investigator	
Carol	Wagner			Medical University of South Carolina	Charleston, SC	Principal Investigator	
John R	Keller			University of Arkansas for Medical Science	Little Rock, AK	Principal Investigator	
Amy	Reynolds			University of Buffalo, Jacobs School of Medicine and Biomedical Sciences	Buffalo, NY	Principal Investigator	
Roberta	Keller			University California, San Francisco	San Francisco, CA	Principal Investigator	
Mark	Hudak			University of Florida College of Medicine	Jacksonville, FL	Principal Investigator	
Adam	Duncan			University of Texas Health Sciences Center	Houston, TX	Principal Investigator	
Ronald	Walshburn			Wake Forest University School of Medicine	Winston Salem, NC	Principal Investigator	
Susan L	Teitelbaum			Icahn School of Medicine at Mount Sinai	New York, NY	Principal Investigator	

Supplemental Online Content: Nonauthor Collaborators
*First name, last name, and suffix (if applicable) are required and will appear in PubMed.

*First Name and Middle Initial(s)	*Last Name	*Suffix (eg, Jr, III)	Academic Degrees	Institution	Location (city, state/province, country)	Role or Contribution, eg, chair, principal investigator	Group (if more than 1 Group listed in the byline) and/or Subgroup (eg, Steering Committee)
Annemarie	Stroustrup			Icahn School of Medicine at Mount Sinai	New York, NY	Principal Investigator	
Andrea	Lampland			Children's Hospital and Clinic	Minneapolis, MN	Principal Investigator	
Dennis	Mayock			University of Washington	Seattle, WA	Principal Investigator	
Jonathan	Mansbach			Boston Children's Hospital	Boston, MA	Principal Investigator	
Jonathan	Spergel			Children's Hospital of Philadelphia	Philadelphia, PA	Principal Investigator	
Michelle	Stevenson			Norton Children's Hospital	Louisville, KY	Principal Investigator	
Cindy	Bauer			Phoenix Children's Hospital	Phoenix, AZ	Principal Investigator	
James	Gern			University of Wisconsin	Madison, WI	Principal Investigator	
Christine	Seroogy			Marshfield Clinic Research Institute	Marshfield, WI	Principal Investigator	
Casper	Bendixsen			Marshfield Clinic Research Institute	Marshfield, WI	Principal Investigator	
Irva	Hertz-Picciotto			University of California Davis Mind Institute	Sacramento, CA	Principal Investigator	
Catherine	Karr			University of Washington	Seattle, WA	Principal Investigator	
Alex	Mason			University of Tennessee Health Science Center	Memphis, TN	Principal Investigator	
Scott	Weiss			Brigham and Women's Hospital	Boston, MA	Principal Investigator	
George	O'Connor			Boston University Medical Center	Boston, MA	Principal Investigator	
Robert	Zeiger			Kaiser Permanente, Southern California	San Diego, CA	Principal Investigator	
Leonard	Bacharier			Washington University of St. Louis	St Louis, MO	Principal Investigator	
Brian	Carter			Children's Mercy	Kansas City, MO	Principal Investigator	
Carmen	Marsit			Emory University	Atlanta, GA	Principal Investigator	
Steven	Pastyrnak			Helen DeVos, Children's Hospital	Grand Rapids, MI	Principal Investigator	

*First name, last name, and suffix (if applicable) are required and will appear in PubMed.

***First Name and Middle Initial(s)**	***Last Name**	***Suffix (eg, Jr, III)**	Academic Degrees	Institution	Location (city, state/province, country)	Role or Contribution, eg, chair, principal investigator	Group (if more than 1 Group listed in the byline) and/or Subgroup (eg, Steering Committee)
Charles	Neal			Kapiolani Medical Center for Women and Children	Providence, RI	Principal Investigator	
Lynne	Smith			Harbour-UCLA Medical Center	Los Angeles, CA	Principal Investigator	
Jennifer	Helderman			Wake Forest University School of Medicine	Winston Salem, NC	Principal Investigator	
Robert	Tepper			Indiana University, Riley Hospital for Children	Indianapolis, IN	Principal Investigator	
Craig	Newschaffer			Pennsylvania State University	University Park, PA	Principal Investigator	
Heather	Volk			Johns Hopkins Bloomberg School of Public Health	Baltimore, MD	Principal Investigator	
Rebecca	Schmidt			University of California, Davis	Sacramento, CA	Principal Investigator	
Jean	Kerver			Michigan State University	East Lansing, MI	Principal Investigator	
Charles	Barone			Henry Ford Health System	Detroit, MI	Principal Investigator	
Patricia	McKane			Michigan Department of Health and Human Services	Lansing, MI	Principal Investigator	
Nigel	Paneth			Michigan State University	East Lansing, MI	Principal Investigator	
Michael	Elliott			University of Michigan	Ann Arbor, MI	Principal Investigator	
Susan	Schantz			University of Illinois, Beckman Institute	Urbana, IL	Principal Investigator	
Christina	Porucznik			University of Utah	Salt Lake City, UT	Principal Investigator	
Robert	Silver			University of Utah	Salt Lake City, UT	Principal Investigator	
Elisabeth	Conradt			University of Utah	Salt Lake City, UT	Principal Investigator	
Michelle	Bosquet-Enlow			Boston Children's Hospital	Boston, MA	Principal Investigator	
Kathi	Huddleston			George Mason University	Fairfax, VA	Principal Investigator	
Nicki	Bush			University of California, San Francisco	San Francisco, CA	Principal Investigator	
Ruby	Nguyen			University of Minnesota	Minneapolis, MN	Principal Investigator	
Emily	Barrett			University of Rochester Medical Center	Rochester, NY	Principal Investigator	
Alison	Hipwell			University of Pittsburgh	Pittsburgh, PA	Principal Investigator	

Supplemental Online Content: Nonauthor Collaborators

*First name, last name, and suffix (if applicable) are required and will appear in PubMed.

***First Name and Middle Initial(s)**	***Last Name**	***Suffix (eg, Jr, III)**	Academic Degrees	Institution	Location (city, state/province, country)	Role or Contribution, eg, chair, principal investigator	Group (if more than 1 Group listed in the byline) and/or Subgroup (eg, Steering Committee)
Kate	Keenan			University of Pittsburgh	Pittsburgh, PA	Principal Investigator	
Christiane	Duarte			New York State Psychiatric Institute	New York, NY	Principal Investigator	
Glorisa	Canino			University of Puerto Rico	San Jaun, PR	Principal Investigator	
Stephen	Dager			University of Washington	Seattle, WA	Principal Investigator	
Robert	Schultz			Children's Hospital of Philadelphia	Philadelphia, PA	Principal Investigator	
Joseph	Piven			University of North Carolina at Chapel Hill	Chapel Hill, NC	Principal Investigator	
Hyagriv	Simhan			University of Pittsburgh Medical Center, Magee Women’s Hospital	Pittsburgh, PA	Principal Investigator	
Frank	Gilliland			University of Southern California	Los Angeles, CA	Principal Investigator	
Shoreh	Farzan			University of Southern California	Los Angeles, CA	Principal Investigator	
Tracy	Bastain			University of Southern California	Los Angeles, CA	Principal Investigator	
