# Supplementary material for: Associations of Neighborhood Opportunity and Social Vulnerability With Trajectories of Childhood Body Mass Index and Obesity Among US Children
Source: JAMA Netw Open. 2022 Dec 22;5(12):e2247957. doi: 10.1001/jamanetworkopen.2022.47957 (PMC9857328; doi:10.1001/jamanetworkopen.2022.47957)
Supplement: Supplement 3. — Data Sharing Statement [file jamanetwopen-e2247957-s003.pdf]

Data Sharing Statement

Aris. Associations of Neighborhood Opportunity and Social Vulnerability With Trajectories of Childhood Body Mass Index and Obesity Among US Children. *JAMA Netw Open*. Published December 22, 2022. doi:10.1001/jamanetworkopen.2022.47957

Data
Data available: No

Additional Information
Explanation for why data not available: Data described in the article, code book, and analytic code will be made available upon request pending application.
